# Abstracts of papers and posters presented at the 2001 Pittsburgh Conference

**DOI:** 10.1155/S1463924601000141

**Published:** 2001


Journal of Automated Methods & Management in Chemistry, Vol. 23, No. 4 (July–August 2001) pp. 99–128

### Abstracts of papers and posters presented at the 2001 Pittsburgh Conference

The following 80 abstracts form Part A of two issues of *Journal of Automated Methods & Management in Chemistry* devoted to abstracts of papers and posters presented this year at the 52nd Pittsburgh Conference, held from 4 to 9 March 2001 in New Orleans, LA, USA. The papers and posters covered a range of topics and techniques, each of which provided valuable information to the conferees and exhibitors alike. Unfortunately, not all of the speakers provided abstracts, so I have been limited to those that were included in the book of Abstracts. This presents a worrying trend, which, added to the disappointing attendance figures, should give the organizing committees food for thought at least. Pittcon is my favourite show and has done a great job for analytical chemistry as a whole. Perhaps people are tiring of New Orleans as a venue, or is it becoming little more than a trade show? Hopefully, it will revive itself when the next show is held from 17 to 22 March 2002, also in New Orleans.

If you need further information about any of these abstracts please contact the authors. It is my intention to publish full papers corresponding to some of these abstracts in future issues of the journal.

Peter B. Stockwell
*Editor*

### Performance criteria and performance characteristics of field-screening test methods

*Kevin Ashley, Ruiguang Song and Paul C. Schlecht, US Department of Health and Human Services, Centers for Disease Control and Prevention, National Institute for Occupational Safety and Health, Cincinnati, OH 45226-1998, USA*

Field-portable test methods may be quantitative, semi-quantitative or qualitative, and screening methods are often used in the field to determine if the concentration of a toxic substance exceeds regulatory or recommended standards or action levels. For on-site analysis, accurate quantitative tests for field measurements may not be available depending on the analyte(s) or specific field situation. Thus, in lieu of more definitive test methods, screening tests based on qualitative or semiquantitative methods are often used to make immediate decisions in the field, e.g. for compliance or risk assessment. Also, quantitative methods may be used for screening purposes in many instances.

To ensure the quality of these screening tests and the decisions made based upon their results, screening methods need to be evaluated with sufficient data and should meet basic performance criteria before their being employed for decision-making purposes. Although quantitative, semiquantitative and qualitative methods demonstrate different characteristics, it is desired that the performance criteria for all three method categories be consistent. If there is consistency, then one can have a sound basis for selecting the most appropriate test(s) for a given application.

To unify the performance criteria for the different types of methods, a performance function is used to characterise both qualitative and semiquantitative methods. In turn, this performance function is related to that for quantitative methods. False-negative rates, false-positive rates, sensitivity and specificity are key characteristics of screening methods that can be determined from the pertinent performance curves. The performance characteristics of each method are related to the uncertainty region associated with each method, and the applicable uncertainty regions can be gleaned from the performance curves. Also, various options for using multiple test results to improve decisions based on test results are provided.

### Performance characteristics and exposure assessment considerations of handheld and field-portable analytical instruments in occupational safety and health

*Perry Logan, Specialty Materials Manufacturing Division, 3M Co., 1400 State Docks Road Building 504, Decatur, AL 35609, USA*

Over the past decade, technology has propelled the use of new portable analytical techniques for the purpose of chemical analysis in the occupational safety and health field. Miniaturisation of chips and circuits has produced handheld computers that now have more computing power than that of mainframe computers only a few decades ago. Smaller size and greater computing power in devices of various analytical technologies has given birth to handheld and field-portable equipment that can be semiquantitative and quantitative in complex matrices and environmental conditions.

These new sensor technologies and field-analytical techniques are helping industrial hygienists and safety professionals perform worker and community exposure assessments more efficiently. However, heavy reliance on a new technology without regard to performance characteristics of the instrumentation could result in overexposures to harmful agents, accidents or wasted resources. In addition, the limitations of each field technology can vary drastically depending on the type of process, agents of interest, environmental conditions, resources available, time constraints and many other factors.

The performance characteristics of the instrument, objectives of the exposure assessment, process and environmental conditions must all be considered when applying http://www.tandf.co.uk/journals
DOI: 10.1080/14639240110087770

any handheld or field-portable analytical instruments. Understanding how an instrument's performance characteristics can be effected by environmental or process conditions is critical when utilising handheld or field-analytical techniques. A systematic approach to evaluate and document all of the necessary information will help ensure that new technologies are utilised properly.

### On-site workshop: performance evaluation of colorimetric air-sampling techniques

*Martin Harper, Department of Environmental Health Sciences, School of Public Health, University of Alabama at Birmingham, RPHB 317, 1530 3rd Avenue S., Birmingham, AL 35294-0022, USA*

One of the simplest and oldest forms of on-site analysis for chemicals is the development of color, which can be compared with standards simply by eye, or by means of an instrumental color comparator, spectrophotometer or densitometer. In air sampling, these devices can be for short-term, near real-time monitoring or for longer-term time-integrated measurements. Their mode of sampling can further be classified as either 'active' (or pumped) or 'passive' (or diffusive).

Depending on the type of sampling, the type of reaction and the type of measurement, the theory of operation leads to linear or semilinear relationships between the measurement (length or depth of stain, absorbance or reflectance) and concentration.

Detector tubes are generally active, near real-time, length-of-stain samplers, although both active and passive time-integrated versions exist. They are normally used for screening measurements and rarely for regulatory compliance purposes, since only a few regulations require near real-time measurements. Their performance is evaluated in accordance with standards published by ANSI (USA) and CEN (Europe). In the USA, this work is undertaken by a third-party certifier (SEI), while in Europe, the manufacturer normally does the tests.

Diffusive samplers are normally long-term, time-integrated samplers. There is a wide variation in analytical finish, including versions that simply change color at a specific alarm dose. Samplers used to demonstrate compliance with regulations or other exposure standards should give results within a specific accuracy range. These ranges are specified in standards in the USA (NIOSH) and Europe (CEN). Several protocols for evaluating the performance of these samplers have been published (NIOSH, CEN, ASTM, ANSI, ISO), but there is no single agency pursuing this validation. Protocols for validating samplers used in environmental monitoring have also been published, but they are unlikely to be used to characterise colorimetric samplers since the sensitivity of the technique does not lend itself to environmental measurement.

The different sampling techniques and evaluation protocols will be described. Particular problems of test comparability and cost will be explored. On-site analysis has advantages of speed and cost. Since there is no need for a sophisticated laboratory, on-site analysis has potential for use in remote areas of the world.

### Automation technology: the new qualification landscape

*Virginia L. Corbin and Richard Andrews, Waters Corp., 34 Maple Street, Milford, MA 01757, USA*

Precision is the watchword when it comes to qualification of laboratory instrumentation and software. Time is the issue—there is never enough!

In the chemical industries, responding to new regulatory requirements and increased competitive pressures has resulted in a technology revolution where equipment performance is an important component to laboratory success.

On average, qualification of a single HPLC system takes between 10 and 12 h for an individual operator to perform. Validating a computer network and its software can take anywhere from 3 to 6 months to a year depending on the size of the system. This means less time for methods development, sample preparation and analysis—the scientist's primary duties.

The principal function of any automated technology is to remove obstacles to the productivity of information people rely on to make decisions. When skilled laborers (e.g. scientists, technicians) are burdened with tasks that rely less on their training as chemists, these activities become perfect candidates for automation.

As technology expands, the demands placed to validate system performance and qualification have increased as well. One innovation, Waters registered trademark, ®, Corporation's Connections Automated Qualification Tools (AQT) trademark, ™, is poised to respond to this critical need. This fully automated, hands-free qualification software provides today's laboratory with an effective, reliable resource for performing system qualification in a fraction of the time of manual processes.

### Software for the development of automated HPLC-integrated sample preparation using column switching

*S. Galushko[1], V. Tanchuk[2], A. Sasko[2] and M. Wltzenbacher[3], [1]Im Wiesengrund 49-b, 64367 Muehltal, Germany, [2]Institute of Bioorganic Chemistry and Petrochemistry, National Academy of Science of the Ukraine, Kiev, Ukraine, [3]Merck KGaA, Frankfurter Str. 250, Darmstadt, Germany*

The objective was to develop software to support the development of fully automated HPLC methods with integrated sample preparation. The application of column switching to sample preparation and analysis has three steps: (1) sample fractionation into the sample matrix and the analytes; (2) transfer of the analytes from the precolumn to the analytical column; and (3) the analytical separation step.

The software guides the chromatographer through all steps of the method development and recommends a precolumn and an analytical column combination, type of an organic modifier, concentrations of an organic modifier for the fractionation and transfer steps both for the precolumn and the analytical column, times for columns switching and a solvent concentration changing.

The software can start guidelines after entering structural formulae of analytes or two initial runs on an analytical column or a precolumn if structures are not available.

The first version of the software supports the method development for the sample preparation using restricted-access media precolumns (LiChrospher RP-4, RP-8, RP-18 ADS) and different analytical reversed-phase columns.

### Membrane-gated microchips for nanoscale preparative biomolecule separations

*Wenju Feng, Tzu Chi Kuo, Lisa Sloan, John Kirk, Jonathan Sweedler and Paul Bohn, Department of Chemistry and Beckman Institute, University of Illinois at Urbana-Champaign, Urbana, IL 61801, USA*

Tremendous advances have been realised in microseparations with the advent of microfabricated separation systems. Minute quantities of biomolecules such as amino acids and PCR products have been separated efficiently in such systems. However, post-separation concentration and manipulation remains a challenge.

We demonstrate the use of nuclear track-etched polycarbonate membranes that can be electrically controlled to capture and release molecules, Le, a molecular gate, combined with an electrophoresis separation channel to collect and manipulate desired components.

A variety of fabrication methods have been explored to obtain such molecular gate-capillary electrophoresis multilayer devices. To this end, glass and polymer materials such as poly(dimethylsiloxane) (PDMS) have been used. PDMS chips incorporating the gate membrane have been sealed by oxygen plasma treatment or by pressure. Both amino acids and DNA restriction fragments have been separated in a glass chip with $<20\,\mu m$ deep by $<100\,\mu m$ diameter channel and detected by laser-induced fluorescence. Samples such as Phi Chi 174 HaeIII restriction fragments have been separated in a coated glass chip filled with hydroxylethylcellulose in a few minutes.

Separations of DNA restriction fragments have also been performed in PDMS chips, and collection of fluorescein by the membrane-gated PDMS chips have been studied. We demonstrate that microfabricated devices not only improve the speed and throughput of electrically driven separations, but also enable the manufacture of multidimensional devices.

Performance characteristics of hybrid separation/collection devices fabricated by these strategies will be discussed.

### Biochip with a microfluidic delivery system for the detection of pathogens

*David L. Stokes, Guy D. Griffin, Minoo Askari and Tuan Vo-Dinh, Advanced Monitoring Development Group, Life Sciences Division, Oak Ridge National Laboratory, Oak Ridge, TN 37831-6101, USA*

This work describes an integrated microfluidic system coupled to a biochip sensor for pathogen detection. The biochip, developed at Oak Ridge National Laboratory, is a $4 \times 4$ or $10 \times 10$ array of independent phototransistors, integrated amplifiers, discriminators and logic circuitry on a single circuit board. The potential for quantitative analysis has been demonstrated with this new photosensor array. Linear dynamic ranges of up to five orders of magnitude have been demonstrated. When used in conjunction with sample amplification techniques (e.g. ELISA or PCR), very low detection limits are possible ($= 10$ organisms).

Furthermore, multiple, specific bioassays can be performed on a chip platform due to the independently functioning detection elements of the array sensor. In this work, a sampling platform (e.g. membrane) is pretreated with an array of capture probes (e.g. antibodies, antigens or DNA probes) and then placed in a watertight sample chamber. An integrated microfluidic system delivers a sample and reagents for the bioassays. With this approach, a specific immobilisation mechanism can be confined to an area corresponding to a single detection element of the biochip. This unique, multifunctional, bench-top device may prove to be an invaluable tool for on-site, rapid analysis for the presence of disease-causing pathogens. It has been applied to the detection of *E. coli*, *B. anthracis* and *L. monocytogenes*. The integration of sample amplification methods such as ELISA and PCR for the detection of very low numbers of organisms with the biochip is discussed.

### Small-volume flow-based immunoassay for interleukin 5 in capillaries and microchips

*Allison N. Phayre, Antonio A. Garcia, James J. Lee and Mark A. Hayes, Department of Chemistry & Biochemistry, Arizona State University, Tempe, AZ 85287, USA*

A small-volume flow-based immunoassay using paramagnetic beads with rare earth magnets, which is renewable, easy to use and adaptable to a variety of biological analyses, is presented.

Antibody-modified paramagnetic beads are flowed through a capillary or microchip and captured by a magnet to form a bed. The analyte of interest is bound to the antibody-modified beads as the sample is flowed through, and a secondary labeled antibody is flowed through and bound for detection (B). This user-friendly assay allows the analysis of nanoliter volume samples and provides flexibility in assay choice, as the target analyte of the assay can be changed simply by changing the antibody-modified beads. Additionally, this assay has the potential to allow a biological sample to be removed from an *in vivo* environment through direct sampling and analysed without intensive sample preparation or manipulation. Enzyme-based and polystyrene bead-based formats on a microchip constitute recent attempts to miniaturise standard immunoassays [1, 2]. However, these techniques are limited by the need for a fluorogenic substrate or a physical barrier from specialised chip

1. Cohen, C. B., Chin-Dixon, E., Jeong S., and Nikiforov, T. T., 1999, *Anal. Biochem.*, **273**, 89.
2. Sato, K., Tokeshi, M., Odake, T., Kimura, H., Ooi, T., Nakao, M., and Kitamori, T., 2000, *Anal. Chem.*, **72**, 1144.

fabrication. Our design allows for small-volume sampling in existing capillary and microchip formats, and is limited only by the availability of antibodies for the analyte of interest. Here we demonstrate this novel approach with interleukin 5 (IL-5), a 23 kDa, 113 amino acid peptide that is a known asthma and inflammatory response marker (A). The capability to assay for IL-5 in nanoliter samples from the asthmatic mouse will be demonstrated and the potential to analyse multiple inflammatory response markers discussed.

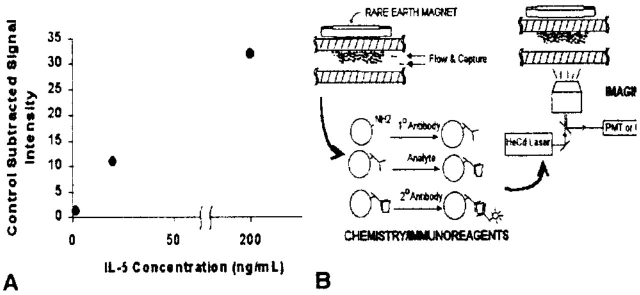

*(A) Preliminary data obtained with IL-5 standards; (B) schematic of flow-based micro-immunoassay.*

### Computer simulation of separation and flow within microfabricated capillaries

*T. Roussel[1], D. Jackson[2], M. Crain[2], R. Baldwln[3], J. Naber[2], K. Walsh[2] and R. Keynton[1], Departments of [1]Mechanical Engineering, [2]Electrical Engineering and [3]Chemistry, University of Louisville, Louisville, KY 40292, USA*

The purpose of this study was to compare the accuracy of a new finite-element software package (Memcad, Microcosm Technologies, Inc., Cambridge, MA, USA) in predicting electrophoretic flow in microchannels to experimental results obtained in microfabricated capillaries. A computational model of the capillary geometry was created from the software version (L-Edit, Tanner Research, Inc., Pasadena, CA, USA) of the actual photolithographic mask used to microfabricate the experimental devices. The dimensions of both the microfabricated and computational model capillaries were 50 pm wide × 15 pm deep × 2000 pm long. In this 'balanced-cross' model, the boundary conditions were (1) the applied voltages at each of the four electrodes corresponding to the actual field strength applied in the CE prototype and (2) a double layer/plane of shear for the fluid at all the walls. During injection, a 250 V $cm^{-1}$ electrical field was applied across the injection capillary to drive the species into the separation capillary. The field was then switched to the terminals of the separation capillary and a finite plug of the simulated injection species was formed and exited the capillary (left of figure) at a speed similar to the CE prototype (right of figure).

The computer simulation yielded a calculated mean velocity of 333 pm $s^{-1}$ as compared with an average plug speed of $372 \pm 13$ pm $s^{-1}$ ($n = 7$) obtained from the experimental devices. As reported in literature [1], electrokinetic focusing was employed to produce a stable plug at the capillary intersection (injection and separation capillary crossing) during the injection phase. This configuration produced a 'pinching' effect of the injected sample at the intersection, which effectively eliminated any premature migration of the species into the separation capillaries and created a denser, more symmetrically shaped plug.

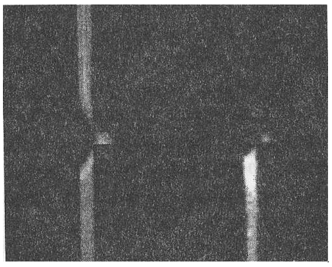

*Simulation (left) and experimental (right) results of plug separation in a microfabricated capillary.*

1. Jacobson *et al.*, 1997, *Anal. Chem.*, **69**, 3212.

### Automated production of natural product extracts

*Bruce E. Richter and Richard E. Carlson, Dionex Corp., SLCTC, 1515 W. 2200 S., Suite A, Salt Lake City, UT 84119, USA*

Interest in the pharmacological active compounds found in plant tissues continues to grow. To identify and study these compounds, they must first be extracted from the tissues in which they naturally occur before applying the various screening techniques. The extraction techniques normally used to remove these compounds from plant tissues require long periods and copious amounts of solvents. In addition, none of these extraction procedures can be automated. Accelerated solvent extraction (ASE) has been proven effective in removing target compounds from a variety of plant tissues. Using ASE, the extraction of compounds from medicinal plants is completed in ~15 min using only 20–30 ml of solvent.

Extraction of the target compounds may be only a part of the isolation process. Interfering compounds such as waxes, pigments and tannins can be co-extracted. These interferences must be removed before the extracts can be subjected to biological or chromatographic screening techniques. Solid-phase extraction (SPE), liquid–liquid extraction (LLE) and preparative liquid chromatography are the techniques most often utilised for removing interferences from plant extracts. However, these steps are separate and not coupled with the extraction process.

This presentation will discuss the coupling of ASE with a liquid-handling apparatus to produce plant extracts free from interfering co-extracted compounds. This combined system produces natural product extracts that are ready for biological or chromatographic assay. The savings in time and cost will be discussed along with the comparison of results from this procedure with those obtained without automation.

### Microchip array-based protein-binding assay using surface plasmon resonance imaging for 'label-free' detection

*Claire E. J. Dentinger, December B. Martin, Krista Witte, Laurence A. Ruiz-Taylor and Peter Wagner, Zyomyx, 3911 Trust Way, Hayward, CA 94545, USA*

We demonstrate a microchip array-based antibody–antigen assay that relies on imaging surface plasmon resonance (SPR) as a means of detecting protein binding. SPR is sensitive to the thickness and index of refraction of material adsorbed at a thin gold film and, for this reason, can detect adsorption at the surface without the addition of a fluorescent or radioactive label. The 'label-free' detection method is a significant advantage as the additional step of labeling the analyte, which is required for detection methods based on either fluorescence or radiolabeling, is particularly cumbersome in high-density protein arrays. For example, fluorescence detection could require labeling thousands of different proteins to perform an assay using a high-density array. Here we describe a simple 'label-free' assay using cytokines immobilised in an array and monitoring antibody–antigen interactions by imaging SPR.

For this work, we have developed a surface that will specifically bind biotinylated proteins and yet is resistant to non-specific protein adsorption. These surfaces consist of a gold substrate coated with a mercaptoundecanoic acid (MUA) self-assembled monolayer onto which biotin-derivatised poly(L-lysine)-graft-poly(ethylene glycol) (PLL-g-PEG-Bx%) is electrostatically adsorbed. The immobilised PLL-g-PEG-Bx% binds streptavidin in a manner that leaves some of streptavidin's binding pockets available for the immobilisation of a wide variety of biotinylated proteins. The MUA, PLL-g-PEG-Bx% and streptavidin layers are characterised with ellipsometry, Fourier transform infrared reflection (FTIR) adsorption spectroscopy, X-ray photoelectron spectroscopy (XPS) and radioactive measurements. Three different percentages of biotin modification (0, 30, 100%) of the PLL-g-PEG, and the amount of streptavidin that binds to each, is investigated. The streptavidin-coated surface is then used to immobilise an array of biotinylated proteins and antibody–antigen binding to this array is followed with the imaging SPR.

### On-site analysis of contaminated surface water

*Karsten Levsen, Fraunhofer Institute of Toxicology and Aerosol Research, D-30625 Hanover, Germany*

River water is used in many countries as a direct or indirect source for drinking water production. Thus, a deliberate or accidental discharge of toxic chemicals into a river may threaten not only the aquatic ecosystem, but also the drinking water supply of a larger population.

Many large rivers are kept continuously under surveillance to identify the release of toxic chemicals during an accident using dynamic biotests. However, these methods do not provide the necessary information on the individual compound released into the aquatic system. Thus, even today, chromatographic methods are still mainly used to monitor individual organic compounds in polluted water. Automated systems based on chromatographic techniques include the actual sampling, analyte extraction and enrichment, chromatographic separation and instrumental detection. Automation for on-site monitoring is most easily achieved if solid-phase extraction (SPE), solid-phase micro-extraction (SPME) or membrane extraction (such as MESI) is used for sample enrichment. Solid phase and membrane extraction can be readily coupled to a chromatographic unit. Depending on the polarity and volatility of the compounds, the on-line enrichment is either combined with gas chromatography (mass spectrometry) (GC(MS)) or with high-performance liquid chromatography (mass spectrometry) (LC(MS)). Volatile compounds in river water can also be analysed by direct coupling of membrane extraction to mass spectrometry (MIMS). On-line SPE-GC(MS) and on-line SPE-LC(MS), as well as MIMS and SPME coupled to GC(MS) are particularly suitable for automated on-site monitoring of polluted river water. As a result of the large mass spectra libraries available, SPE-GC/MS and SPME-GC/MS and also MIMS are suitable for non-target screening of polluted surface water, but equally well suited for quantitative target analysis. Non-target screening of polar compounds is more difficult with SPE-LC/MS. Thus, this very powerful hyphenation is mainly used for target analysis, such as pesticide analysis.

The use of special sorbents in on-line SPE-GC/MS and in on-line SPE-LC/MS allows the specific enrichment of target compounds or compound classes. Such an increase in selectivity is achieved using immuno-affinity sorbents or molecular imprinted polymers, which can also be applied with SPME. For ionic compounds, ion-exchange sorbents are available. Furthermore, the problem of humic acid interferences can be overcome using restricted access material (RAM), which allows the exclusion of high molecular weight compounds.

Again, these new adsorbents are well suited to automated on-site analysis of polluted surface water.

### Field air analysis with SPME

*Jacek Koziel[1,2], Fabio Augusto[1,3], Claudia Zini[1], Mingyu Jia[1], Abir Khaled[1], Japheth Noah[1] and Janusz Pawliszyn[1], [1]Department of Chemistry, University of Waterloo, Waterloo, ON N2L3G1, Canada; current addresses: [2]Texas Agricultural Experiment Station 2, Texas A&M University, Amarillo, TX 79106, USA, [3]IQ-Unicamp, CP 6154—130830970 Campinas, São Paulo, Brazil*

Solid-phase micro-extraction (SPME) presents many advantages over conventional analytical methods by combining sampling, preconcentration and direct transfer of the analytes into a standard gas chromatography (GC). Since its introduction in the early 1990s, SPME has been successfully applied to the sampling and analysis of environmental samples. Besides a routine laboratory analysis, SPME can be used in field air sampling and on-site analysis. The on-site analysis with SPME allows the immediate assessment of sampled air, increases sample throughput, does not require sample preservation and allows 'hot spot' sampling for many volatile organic compounds (VOC). This paper presents an overview of the current methods for quantitative air sampling and analysis with SPME using both grab and time-weighted average (TWA) modes, for short- and long-term exposure assessment. New developments in spot, rapid and long-term sampling will be illustrated with field data from indoor air surveys, trace biogenic and aroma studies, and screening for particulates in vehicle exhausts. Advantages and challenges associated with field air analysis with SPME will also be discussed.

### Field measurements of atmospheric trace gas and particle composition

*Purnendu K. Dasgupta, C. Bradley Boring, Rida Al-Horr and Genfa Zhang, Department of Chemistry and Biochemistry and The Institute of Environmental and Human Health, Texas Tech University, Lubbock, TX 79409, USA*

The science of real-time or near real-time measurement of atmospheric gases and particles is still in its infancy. After several iterations over the last 10 years, we have arrived at the present design for the near real-time measurement of ionogenic-soluble atmospheric gases (this includes all acid gases and ammonia) and the soluble ionic constituents of atmospheric particulate matter. Although the approach and exact design continues to evolve, the present design is particularly robust and versatile. Soluble gases are removed with a parallel plate denuder with microstructured surfaces made from Plexiglas® that renders them wettable. The plates are wetted with a continuous flow of dilute $H_2O_2$ solution. The liquid effluent from the denuder is concentrated on one of a pair of sequential cation and anion-exchange columns. Every 15 min, sampling is switched from one pair of columns to the other.

Before elution, each column is washed in-line with water. The cation exchanger is eluted with NaOH and the liberated $NH_3$ measured conductometrically by passage on one side of a microporous membrane with water flowing on the other side as a receiver. The anion preconcentration column is part of an anion chromatography system and conventional anion chromatographic analysis is carried out.

The gas phase effluent from the denuder contains the aerosol. A specially designed valve directs this stream of one of two 25-mm glassfiber filters. Again, sampling continues for 15 min until the filter is switched for the other. The freshly sampled filter is washed with water for 8.5 min and the washings preconcentrated on a sequential cation/anion column exactly as in the gas analyser portion of the system. For the next 6.5 min, the filter is dried with clean, hot air so it is ready to be switched to begin sampling at the end of 15 min. LODs for both gaseous and particulate analytes are in the low to sub-ng $m^{-3}$ range. Atmospheric measurement data from 1999 Atlanta and 2000 Houston Supersite campaigns will be presented.

### Membrane introduction mass spectrometry (MIMS) for on-site analysis

*R. Graham Cooks, Leah Riter, Zoltan Takats, Kim Koch, Garth Patterson and Zheng Ouyang, Chemistry Department, Purdue University, West Lafayette, IN 47907-1393, USA*

Membrane introduction mass spectrometry is a method by which volatile and semivolatile compounds in aqueous and, less commonly, air samples can be identified and quantified down to low levels. The analytes partition into the membrane, typically a silicone polymer, and diffuse through it before being released into the (tandem) mass spectrometer. A strength of MIMS is that it requires little or no sample preparation. MIMS is also well suited to on-line analysis but has seen limited application in this area because of the size, complexity and relatively low reliability of mass spectrometers. The method, including a number of recent variants, has been reviewed [1].

This paper describes new membrane introduction systems that further improve detection limits of MIMS experiments. It also describes a miniature ion-trap mass spectrometer that is much more appropriately scaled to MIMS use than current commercial instruments. Finally, initial applications of the MIMS/miniature cylindrical ion-trap combination are described. These focus on the detection of chloramines in water and nitro-aromatics and dimethyl methylphosphonate in air.

Miniaturisation of mass spectrometers is currently a popular objective among instrument builders. The ion-trap, especially in the form of the cylindrical ion-trap (CIT), lends itself to miniaturisation and to the assembly of arrays of miniature analysers. CITs of just a few millimeters internal radius are shown to perform well as mass spectrometers, giving unit mass resolution over a range of several hundred mass units. MIMS is shown to be a suitable method of sample introduction into these instruments.

Two innovations in the membrane introduction system are described. In the first, known as single-sided MIMS, less volatile compounds are examined by a trap-and-release process in which they are partitioned into but not required to diffuse through the membrane. Instead, they are thermally released from the same side. This method is analogous to *in situ* solid-phase micro-extraction or, if the membrane is chemically derivatised for increased selectivity, to affinity chromatography. The second method employs a membrane with much higher surface area than commonly used. This method is shown to give low ppt detection limits for a variety of compounds of environmental interest in air samples.

1. Johnson, R. C., Cooks, R. G., Allen, T. M., Cisper, M. E., and Hemberger, P. H., 2000, *Mass Spectrom. Rev.*, **19**, 1.

### Calibration of sampling/sample preparation techniques for on-site analysis

*Janusz Pawliszyn, Department of Chemistry, University of Waterloo, Waterloo, Ontario N2L 3G1, Canada*

The ultimate goal of the chemist is to perform analysis at a place where a sample is located rather than moving the sample to laboratory, as it is common practice in many cases at present. This approach eliminates errors and reduces the time associated with sample transport and storage and, therefore, results in more accurate, precise and faster analytical data. In addition to portability, three other important features of ideal field sample preparation technique are the elimination of solvent use, integration with a sampling step and convenient calibration, preferably without the use of standards during on-site operation.

We have been involved in fundamental investigations of extraction processes in a number of practical systems. Such geometries as coated fibers [1–3] and membranes [4] in open- and closed-bed configurations and exhaustive, micro-extraction, permeation as well as time-weighted average (TWA) approaches were studied. During these investigations, simple correlations have been identified among physicochemical and geometric parameters associated with sampling/sample preparation systems and the accumulation rate and/or amount of analyte collected on the extraction phase. For example, the quantity of analytes extracted for the micro-extraction technique at equilibrium is related to the extraction phase/sample matrix distribution constant. This constant can be found using calculation methods, or it can be stored in the memory of the instrument before the deployment in the field. If the extraction phase is exposed only for a short period to the investigated system, then the amount of analyte accumulated onto the extraction phase is related to the diffusion coefficient in the sample matrix.

Again, this parameter can be found before field measurement. In the case of extraction of analytes through the membrane, the rate of permeation is related to the product of diffusion coefficient in the membrane material and the membrane material/sample matrix distribution constant. Several simple on-line approaches to measure these parameters, and, therefore, to calibrate the systems in the field, will be discussed. Examples of field applications will be given.

1. *Solid Phase Microextraction: Theory and Practice* (New York: Wiley-VCH, 1997).
2. Khaled, A., and Pawliszyn, J., 2000, *J. Chromatogr.*, **892**, 455.
3. Koziel, J., Jia, M., and Pawliszyn, J., 2000, *Anal Chem.*, **72**, 1064.
4. Luo, Y., and Pawliszyn, J., 2000, *Anal. Chem.*, **72**, 1064.

### Analysis of combinatorial MS data using automated molecular fragmentation assignment

*Antony J. Williams[1], Vitaly Lashin[2] and Ilya Troitskiy[2], [1]Advanced Chemistry Development, 90 Adelaide Street West, Suite 702, Toronto, Ontario M5H 3V9, Canada, [2]Advanced Chemistry Development, Russian Office, 6 Bakuleva Street, Moscow 117513, Russia*

The analysis of combinatorial plate MS data has generally been approached using searching of the molecular mass of the parent ion based upon an input list of expected molecular masses. This approach leaves the analysis open to misinterpretation since any particular molecular mass can, of course, give rise to many chemical structures. Even when restricted to include particular fragments based on the input starting materials, there are numerous final isomeric structures that can give rise to a match.

The primary intention of the combinatorial mass spectrometry technique is for high throughput. For final analysis of products, generally a combination of both NMR and MS spectroscopy is applied for structure elucidation. It is suggested that acquisition of MS data using ionisation to induce fragmentation does give rise to significantly more data to confirm *structure* rather than simple molecular mass. Such an approach would be of value if software tools were available that could quickly analyse in a facile manner the experimental fragmentation with that expected for the suggested chemical structure. We introduce here software that provides a means to analyse 96-well plate MS *data* acquired with fragmentation.

The software utilises rules-based fragmentation algorithms to generate the theoretical mass spectrum and a matching algorithm that compares the experimental and theoretical fragmentation spectra. The connectivity of such tools to an integrated molecular structure-handling capability including molecular fragmentation is essential for today's structure-based directives.

### Speciation and identification of selenium metabolites in human urines

*Tiffany H. Cao, Michelle M. Woznichak, Sheldon W. May and Richard F. Browner, Georgia Institute of Technology, Atlanta, GA 30332-0400, USA*

The biology and biochemistry of selenium are subjects of intense current interest, particularly from the viewpoint of public health. Selenium has long been recognised as a 'dietary antioxidant' and recently as an anticarcinogenic agent. Selenium deficiency has been linked to heart diseases, arthritis, cancer and, recently, AIDS.

Recent large-scale human studies confirm that selenium supplementation reduces the incidence of prostate, colorectal and lung cancers. While seleno-enzymes and selenium-binding proteins have attracted much recent attention, it is now clear that the antitumorigenic effects of selenium must arise, at least in part, from mechanisms involving low molecular weight selenium metabolites. However, the pathways and mechanism of selenium metabolism that lead to these beneficial effects are poorly understood. Very little evidence exists about selenium metabolites are *actually generated* in human selenium metabolism. Therefore, the overall objective of this project is to obtain structural information about the selenium-containing metabolites that are actually generated in human metabolism. Traditionally, extremely low concentration in biological samples, insufficient knowledge of chemical structure, lack of available standards and thermal instabilities of organoselenium compounds have hindered the progress of selenium identification in complex biological matrices.

This paper presents the speciation of some organoselenium compounds found in human urine samples using high performance liquid chromatography/inductively coupled plasma/mass spectrometry (HPLC/ICP-MS) with an oscillating capillary nebuliser interface. The identification was done using electrospray-MS/MS. Preliminary studies have demonstrated the ability to detect selenomethionine, which had been added into NIST urine at the 100 ppb level. Moreover, we have successfully identified two Se metabolites in human urine, selenomethionine and selenocystamine, using reversed-phase chromatographic separation (see the figure). This is the first time, to our knowledge, that these two selenium metabolites have ever been detected in human urine.

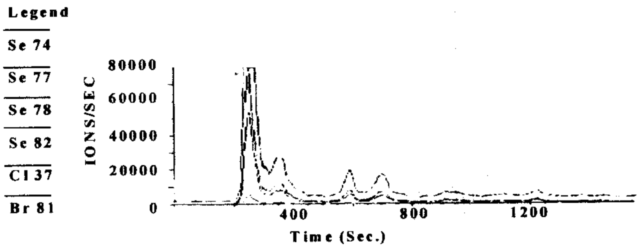

*HPLC/ICP/MS chromatogram of selenium-containing compounds in 50 pl concentrated human urine.*

### LIMS and e-business

*Kim Ryals and Fred Rider, Accelerated Technology Laboratories, Inc., 496 Holly Grove School Road, West End, NC 27376, USA*

With the phenomenal growth of the World Wide Web (WWW), it is natural for laboratories to want to offer their services along side other e-businesses. The three main designs to provide this include: Web Application Provider (WAP); Web application; and Web-enabled Laboratory Information Management System (LIMS).

A WAP 'rents' a database that is available to the laboratory via the WWW. The database is partitioned for each laboratory and the WAP maintains the database.

A Web Application is a LIMS run on a laboratory's own website and is administered by the laboratory. The database may reside in the laboratory or with an Internet Service Provider (ISP).

A Web-enabled LIMS is an internal database with links to the WWW. The users in the laboratory interact with the database either via an Intranet or with a conventional Local Area Network (LAN) application.

Because each laboratory is unique, one solution does not fit all laboratories. Each configuration has its own strengths and weaknesses in areas such as security, data integrity, privacy, administration and flexibility. This presentation will explore the strengths and weaknesses of each design and provide recommendations for laboratories to design, implement and profit from e-business.

### Automation tools for a chromatography data system

*John T. Brady, Gerald Lisowski and Soheil Saadat, Scientific Software, Inc., 6612 Owens Drive Pleasanton, CA 94588, USA*

This presentation will focus on the ability to modify and or input/output data in a chromatography data system (CDS). This can be done using supplied automation tools. These tools support either Visual BASIC Scripting, Visual BASIC or C++ programming languages. One application of this automation tool is to simplify the user interface.

An interface can be designed so the user inputs minimal information, the rest being generated by the system. Another application is after a large sequence is run, a program can be used to reprocess samples in defined groups with additional functions performed such as subtracting a blank from a sample and presenting a report to a LIMS system. Other applications have been designed for the inputting or extraction of parameters from the software to third-party software such as LIMS or other databases.

This presentation will demonstrate these functions using Scientific Software, Inc.'s, EZChrom *Elite* Chromatography Data System.

### Impact of implementing LIMS on laboratory efficiency

*Mohamed M. Gohar, Ohio Arson Crime Laboratory, Division of State Fire Marshal, 10233 N. Crosset Hill Drive, Pickerington, OH 43147, USA*

The use of Laboratory Information Management System (LIMS) integrated with data acquisition hardware and software has significantly improved the efficiency and quality of data in analytical laboratories. As this approach becomes the core of modern laboratories, several issues affecting selection and configurations will assure successful implementations.

This paper looks at the automation history of the State of Ohio Arson Crime Laboratory showing migration path over the past 25 years from the oldest to the newest: how we selected hardware and software, the importance of the right integration of different components, the learning curve for system managers and users, and security of databases.

During 1980, with a staff of nine employees, it took our laboratory >100 days on average to report a non-rush case and 22 days for a rush case. Progress was made by hiring additional personnel. From 1984 to 1986, the turnover time (TOT) for rush cases dropped to 7 days and the non-rush cases dropped to 20 days. Those figures were very impressive at that time and were achieved by utilising the service of 11–14 laboratory staff members. Since the full implementation of LIMS, the average TOT has been 3 workdays from 1989 to 1999. Average TOT for May 2000 for rush or non-rush cases is 2 workdays. These figures were achieved with a total laboratory staff of six employees. The quantitative measurement of the impact of using automation on the efficiency of laboratory management and data quality will be graphically presented.

### Exploiting developments in business-to-business exchanges to increase laboratory efficiency

*Kevin P. Smith[1], Tony Johnson[1] and Clive Higgins[2], [1]Thermo LabSystems, 1 St George's Court, Altrincham, Manchester WA14 5TP, UK, [2]Thermo LabSystems, Inc., 100 Cummings Center, Suite 407J, Beverly, MA 01915, USA*

The majority of scientists will be well aware of the usefulness of the Internet as a medium for enhanced knowledge management. However, the Internet also brings a number of other benefits, specifically in the area of business-to-business e-commerce. We have been exploring ways that laboratories might link their internal systems (LIMS, Purchasing, Quoting/Invoicing, Reagent/Consumables Tracking, Customer Relationship Management) with external supplier and customer systems. It is clear that there are opportunities to increase efficiency by streamlining the processes involved in such transactions.

The paper will explain some of the technology employed, much of which is based around industry standards such as XML. It will then give some real-world examples of business-to-business successes in the laboratory environment.

### Automated *in vitro* drug delivery and functionality test for electrotransport transdermal systems

*Hong Gao, Huiming Lin, Erik Scott and Steven Fields, ALZA Corp., 1900 Charleston Road, Mountain View, CA 94039, USA*

Electrotransport systems incorporate a technology unique in the transdermal drug-delivery field. A highly controlled amount of drug can be delivered into the skin. The amount is regulated by the design of the current profile and the electrotransport mechanism.

An Electrotransport System (ETS) consists of an electronic component (battery, control circuitry and actuator) and a drug reservoir. During the product development and evaluation processes, *in vitro* drug delivery from the ETS is a key measure of its functionality and reliability. In this delivery test, a major challenge is that the ETS cannot be submerged in a receptor media as is routinely done for passive delivery transdermal systems. In addition, the delivery test system needs to record all of the electronic data that profile the ETS performance.

An automated system functionality test has been developed and used to evaluate an ETS system designed for the administration of fentanyl for acute pain. The test can measure dose current, dose duration and diagnostic voltage while the drug is delivered and collected for analysis by HPLC. The system functionality test apparatus consists of a fixture, sample collector unit, and computer data collection and process system. Six systems can be placed on the fixture at same time. A dose actuator automatically initiates multiple dosing of the systems according to the programmed protocol. The released drug then is collected with a receptor buffer. At the same time, dose current, dose duration and diagnostic voltages are monitored in real time, with data processing performed after the test.

The data collected for hundreds of electrotransport systems over 3 years indicate that this highly automated system is very reliable and consistent. The amount of drug delivered is well correlated with applied current.

### New automation concepts for dissolution tests

*Rolf Rolli, Sotax Ltd, Allschwil, Switzerland*

For many years, automation concepts for dissolution laboratories are discussed and solutions realized. At the beginning, the focus was more on the hardware side and its ability to meet all needs of the laboratory. Today, validation of the systems has become the highest priority. Comprehensive systems have a very high level of complexity and validation takes considerable time. Thus, automation concepts for specific tasks are realized to give a high level of operational security as well as ease of validation.

New specific solutions for automation of dissolution testing with semi-automated and fully automated system are described. In selecting an ideal solution for automation, a number of criteria have to be considered.

- Quantity of tests per year.
- Test duration and sampling points.
- Medium and pH-changes with solvent-addition and/or full change.
- Standard monitoring.
- Pellet testing.
- Sinkers.

Semi-automated systems are based on a modular concept allowing one to customize and provide UV on-line, HPLC on- or off-line solutions with all options like solvent replacement after sampling and solvent addition for pH change.

A fully automated test instrument is the most promising solution for a higher number of short-time tests and sampling points. This solution offers to test up to 15 completely different USP 2 tests in series. With such a system, all steps are fully automated, from medium preparation, tablet input to the printout of the reports, and it includes a very efficient cleaning device that prevents any carry over from test to test. Test-requiring baskets are handled with the Basket-Station. This system allows up to 10 USP 1 tests to be analysed.

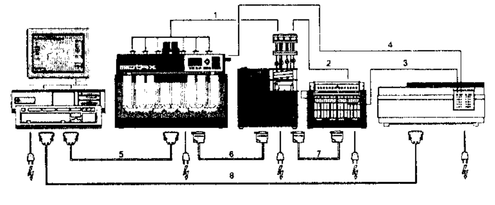

*Semi-automatic on–off-line system.*

### Advances in fiberoptic dissolution testing

*Kevin C. Bynum, Abe Kassis, Lane Gehrlein and Philip Palermo, Purdue Pharma L. P., International Research and Development Center, 444 Saw Mill River Road, Ardsley, NY 10502, USA*

An *in situ* fiberoptic dissolution system has been developed for the analysis of dissolution profiles. The system utilises 12 immersion probes to acquire UV spectra during a dissolution test. Each probe is placed inside a dissolution vessel and remains in the vessel for the duration of the test. Each probe is then coupled to a spectrometer by means of a fiberoptic light guide. Twelve PDA spectrometers are used to collect an absorbance spectrum from each probe, at set time intervals, during the dissolution test. After set-up, the system runs without analyst intervention for up to 72 h. All results are automatically calculated and secured in real time.

This publication presents data from a number of pharmaceutical dosage forms generated in a production environment. It will discuss the application of ATR probes to dissolution testing. The use of a 'probe in shaft' sampling technique will also be compared with more traditional sampling mechanisms. Finally, the simultaneous real-time determination of dissolution rate, assay, identification and content uniformity will be discussed. The validation of the system and software will also be presented.

### Bringing the full power of Matlab™ to on-line process spectroscopy

*Bruce McIntosh, Shih-Ying Chang, Larry McDermott and Larry Dingle, Orbital Sciences Corp., 2771 N. Garey Avenue, Pomona, CA 91767, USA*

Matlab has become the preferred environment for developing advanced chemometric algorithms, both in academic and industrial research. Often, however, there has been substantial delay between the method development and its availability as a tool for on-line predictions with process analysers. This delay restricted the usage of Matlab to being a research tool only. On-line analyser users could not directly access the benefits of new algorithms and models until the developers of commercial chemometrics packages updated their products to use them. Since these packages are large and complex, updates to add new features are infrequent. A final problem with this approach is that providing the algorithm to a third party to incorporate normally will release it to all users of the package. When industrial R&D dollars are spent for algorithm development, this may not be all that desirable.

This paper will describe a set of tools that can be used fully to implement the Matlab environment on an FTIR-based on-line process analyser. We have developed two software components. The first is a program that facilitates the input of proprietary spectral data into Matlab for model building. The second and more important component is an interface to allow Matlab to act as a run-time computational server to perform on-line chemometric operations automatically. Matlab is passed the name of an M script detailing the prediction process, the name of a spectrum to process and the name of an ASCII file to create with the results of the predictions. Our standard process-monitoring software then uses these results in the same way they do predictions from proprietary packages.

We will show examples of on-line predictions for monitoring refinery blending using conventional PLS and PCR models along with predictions using an advanced version of PLS and locally weighted regression. We will also address the advantages of applying those Matlab-based advanced chemometric tools for on-line applications.

### Hardware and software for process analysis and control via FT-NIR

*Steve De Jesus, Jon Goode, Sameer Londhe and Qian Wang, Bruker Optics, 19 Fortune Drive, Billerica, MA 01821, USA*

The successful implementation of an FT-NIR spectroscopy system for the analysis and control of a production line depends on a broad array of factors. Such issues as the accuracy and repeatability of the spectral analysis are only the tip of the iceberg. A successful process application must include the right hardware and software choices to facilitate the desired process analyses and to provide the level of control required. Flexible interfacing options are needed in several areas of this type of implementation including the spectrometer-to-sample interface, the user-to-equipment interface and the system-to-system interface between the spectrometer and the control system.

Through examination of several real-world examples, elements of successful FT-NIR process analysis and control systems will be considered in terms of equipment and software features that facilitate integration into existing plant control systems. The evolution of such systems from initial trial runs in the plant to fully operational process control systems will be illustrated.

### Decentralised process gas chromatographs: requirements and benefits

*Ulrich Gokeler[1] and Friedhelm Mueller[2], [1]Siemens Applied Automation, 7101 Hollister Road, Houston, TX 77040, USA, [2]Siemens AG, 76181 Karlsruhe, Germany*

On-line process gas chromatographs are typically concentrated on several locations throughout a production plant in individual or common analyser shelters due to their complexity, performance requirements and maintenance. Consequently, not only is it necessary to build analyser shelters, but also the samples to these centrally located analysers have to be piped from the sampling points to the analyser shelters and back without changing the sample integrity, which often by itself can be a major challenge. To minimise the number of analysers and the associated infrastructure costs, most of the analysers are responsible for a number of individual sample streams that are analysed in sequence. Nevertheless the investment associated with analysers, shelters and sample supply lines is rather significant.

The concept of decentralising process gas chromatographs is the utilisation of small individual analysers inside the process unit, directly at or next to the sampling point. Each analyser may only be responsible for one or two sample streams located very close to the analyser, and thus has consequently a much faster result update than a multistream analyser. It also has much lower installation costs because of the lack of required analyser shelter. Because of the location of the sampling points, the number of decentralised analysers will typically be higher compared with the number of analysers in shelters because each analyser is only responsible for one or two sample streams.

Naturally, such a process gas chromatograph design has to have certain features that are fundamentally different when compared with traditional on-line process gas chromatographs, and consequently the miniaturisation of existing hardware alone will not satisfy the practical needs. This type of analyser has to be very compact and suitable for the process environment. It also has to have a modular design for very easy interchangeability concerning analyser hardware and must be significantly cheaper per sampling point.

Ideally, the reward could be simple, low-cost analysers that provide faster result update, have very few maintainable items and are easy to repair. This analyser could then be installed at the sampling points, needing only a simple sample preparation system with very short sample lines and would not require an analyser shelter.

This presentation will discuss the concept and the requirements of decentralised process gas chromatographs, possible suitable applications, analytical requirements as well as possible analytical and financial benefits.

### Building confidence in on-line and QC chemometric-based analysers

*Larry McDermott, Martha Ranc, Bruce McIntosh and Shih Ying Chang, Orbital Sciences Corp., Applied Instrument Technologies, 2771 N. Garey Avenue, Pomona, CA 91767, USA*

Process infrared and near infrared analysers are continuing to gain acceptance in a wide variety of industrial applications. These systems are 'secondary' methods based on chemometric correlations to conventional analytical methods. These systems are dramatically different from conventional sensors and transmitters that plant personnel are familiar and comfortable with.

These systems also provide a wealth of information that plant personnel are not used to having accessible in real-time. This paper will discuss the implementation of a number of steps and procedures to build confidence in new process analysers. A core component of this approach is the use of control charts for numerous system and analytical level variables such as those recommended in the ASTM Standard Practice for the Validation of Multivariate Process Infrared Spectrophotometers. In addition, model and instrument validation tools are applied to each prediction, providing operators with confidence statistics. All of the recommended steps of this practice can be automatically implemented in an on-line system to build plant confidence in the analytical results. The ASTM practice calls for the calculation and display of basic photometric and predicted variables including the wavelength stability, photometric noise, baseline stability, spectral resolution photometric linearity, toluene PCR model statistics, and property prediction models statistics including the predicted value, Mahalanobis distance and spectral residual. This paper will discuss the implementation of a system validation protocol and it presents examples from on-line systems.

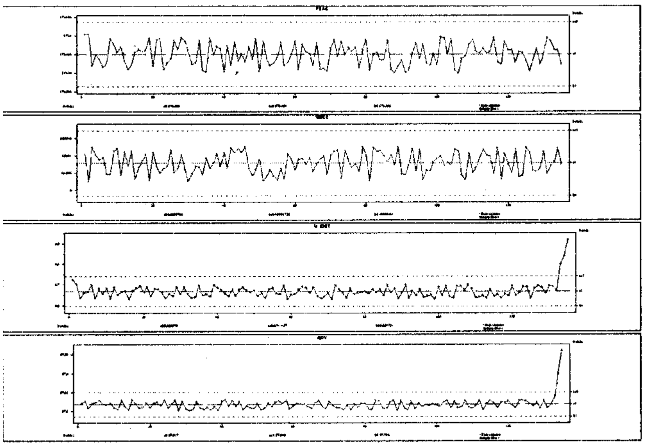

*Example of process analyser control charts for peak center, instrument noise, Mahalanobis distance and RON.*

### Miniaturization for chemical analysis and synthesis

*Andreas Manz, Imperial College of Science Technology and Medicine, AstraZeneca/SmithKline Beecham Centre for Analytical Sciences, London SW7 2AY, UK*

Since diffusive mass transport plays a key role in both the separation and mixing of molecules, a miniaturization of any known set-up results in higher speed. The time-scale goes with the square of the scaling factor, i.e. for a 10-fold decrease in $x$, $y$ and $z$, a 100-fold faster process can be observed. This has been shown for electrophoresis and other separation techniques. To a lesser extent, chemical synthesis has been performed at a small scale. Thermodynamic barriers can ultimately stop the speeding-up.

Interestingly, however, a surprisingly large number of C—C bond-forming reactions can be carried out on the micrometer scale and with good yields! We have used a silicon microchip for the Wittig and Ugi reactions. In both cases, near 100% yields have been observed in $<1$ s. This makes it a very attractive approach to drug discovery chemists.

In addition, the same chip has been used for bioassays. Horseradish peroxidase assays could be done with 'incubation time' of only a few hundred milliseconds.

If now the time-scale is 100 times shorter and the volumes are 1000 times smaller, there might be a detection limit problem. Therefore, fluorescence has been the main detection scheme to date. I will show a few examples of other detectors, like potentiometric sensors, electrochemiluminescence, mass spectrometry and plasma emission spectroscopy.

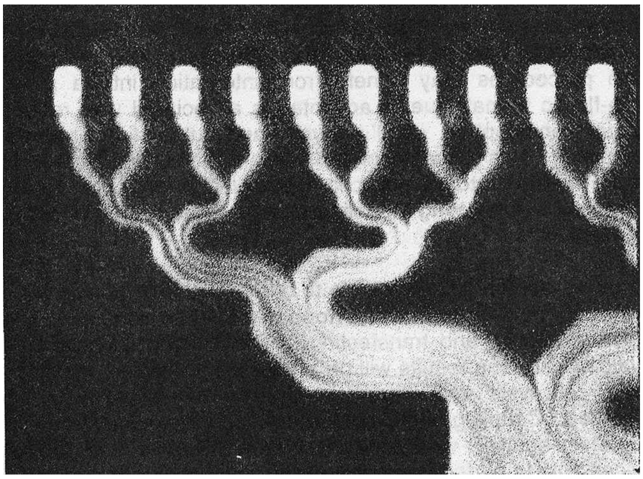

scientific and commercial interest in these devices has waned.

In contrast, the past decade has seen interest in microfabricated fluidic systems grow tremendously. These miniaturised fluidic systems have more closely paralleled the benefits we have realised in the miniaturisation of microelectronics, i.e. small, low-cost devices that deliver *increasing* performance. Microfluidic systems have shown the ability to integrate chemical processing and reactions with chemical analysis. The results have been automated systems that process picoliter scale volumes with high-performance. Such systems have applicability to a broad scope of chemical and biochemical measurement problems such as *in-situ* monitoring, field analysis, clinical diagnostics and high-throughput experimentation. Success in microfluidics devices has renewed interest in miniaturisation of other chemical measurement technology such as mass spectrometry, NMR and ion mobility spectrometry. Prospects for the field of miniature chemical systems will be discussed.

Research was sponsored by the Office of Research and Development, US Department of Energy, Contract No. DE-AC05-00OR22725 with Oak Ridge National Laboratory, managed and operated by UT-Battelle, LLC.

### Lab-on-a-Chip technology: applications in life science

*Monika Dittmann[1], Meike Kuschel[1], Odilo Mueller[1], Stephane Mouradian[2] and Bob Dubrow[2], [1]Agilent Technologies, Waldbronn, Germany, [2]Caliper Technologies Corp., Mountain View, CA, USA*

A Lab-on-a-Chip system developed by Agilent Technologies and Caliper Technologies can be employed for a variety of rapid automated analyses in the life sciences. These are, for example, analyses of PCR fragments, RFLP fragments, total RNA, mRNA and a wide range of protein samples. The system consists of disposable glass chips, optimized reagent kits, computer-controlled instrumentation, as well as an intuitive and user-friendly software for automated data analysis.

The advantages of Lab-on-a-Chip technology compared with conventional gel electrophoresis are demonstrated for the size and quantitation analysis of nucleic acids and proteins of various origins. The most prominent advantages are good sizing and quantitation accuracy and reproducibility, fast analysis times, reduced manual labour, and automated data analysis.

It is demonstrated how Lab-on-a-Chip technology can be successfully employed in many areas of molecular biology (optimization of PCR reactions, performance optimization of DNA arrays, quality control of total and mRNA preparations) as well as biochemistry (expression studies, quality control of protein preparations, purity check of protein column fractions).

### Miniaturised analytical systems: how wide—how deep?

*J. Michael Ramsey, Chemical and Analytical Sciences, Oak Ridge National Laboratory, PO Box 2008, Oak Ridge, TN 37831-6142, USA*

Truly miniaturised chemical instrumentation and systems have been discussed for a quarter of a century. The first such device was a microfabricated gas chromatograph that was fabricated on a 5-cm diameter silicon wafer. While this work was seminal to the area, it did not deliver a miniature device with performance commensurate with laboratory-scale instrumentation and hence

### Automated on-line monitoring of VOC at the PPT level

*Franck Amiet and Pascale Baumard, Chromato-Sud Co., 15 route d'Artiguelongue, F-33240 Saint-Antoine, France*

Because of increasing pollution levels and their impact on people's health, more programs are developed aimed at monitoring atmospheric pollution. Some of the pollutants have been quantified on a routine way for several years. However, the analysis of volatile organic compounds (VOC) on an automated way is still a challenge.

The airmOzone rack offers the possibility to monitor continuously the wide range of ozone precursors down to ppt levels. As the system is constituted of two gas chromatographs (4U), each of the units is dedicated to the analyse of a specific range of compounds.

The choice of adapted analytical columns makes it possible to quantify each compound from C2 to C10 individually. The sample is not prepared in any way (not dried), and the injection is splitless. Thereby, the analyse of polar compounds is possible.

The software controlling the system is very easy to use. It is possible to program different methods and, therefore, to check regularly the calibration and good operating conditions of the system automatically. It is, therefore, easy to introduce quality-control procedures such as a validation of the results by regular calibration runs. Finally, several options for air and hydrogen generators allow one to finish the rack. In this configuration, the system is fully automatic with remote control.

Several European measuring networks are generating results from the airmOzone rack. Results of calibration runs and of on-line field samples are shown in the presentation.

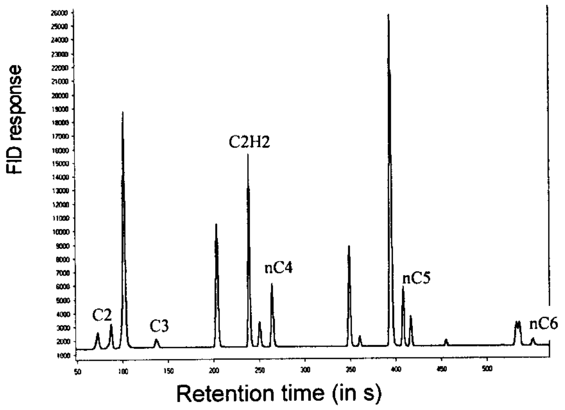

*Standard compound chromatogram (02–06 range).*

### Rapid-response continuous emissions monitor for metals in stack gases

*Geoffrey N. Coleman[1] and Michael D. Seltzer[2], [1]Thermo Elemental, 27 Forge Parkway, Franklin, MA 02038, [2]Chemistry and Materials Branch, Code 4T4230D, Naval Air Warfare Center—Weapons Division, China Lake, CA 93555, USA*

Continuous monitoring of hazardous air pollutant (HAP) metals in stack gases emitted by demilitarisation furnaces, hazardous waste incinerators, municipal waste incinerators, medical waste incinerators, etc. both provides documentation of compliance with regulations and permits optimisation of combustion and pollutant removal processes. The ability to measure with a response time of <2 min facilitates regulatory compliance through closed-loop process control: waste feed rates can be automatically adjusted to prevent permit exceedences. Clearly, the response time of the system impacts the efficacy of the process control arrangement. Consequently, control limits must be lower for less-rapidly responding systems to ensure that transient events do not lead exceedences and the consequential penalties.

The essentially turnkey instrument system is based on an axially viewed argon ICP, which provides for the determination of the 14 hazardous air pollutant elements, as well as many other elements which may be of process monitoring interest.

One development challenge is the introduction of air into an argon ICP at a rate consistent with sensitivity and torch longevity requirements. Another is sample collection and transport from both dry stacks, where pollution control consists essentially of only a bag house and temperatures may reach 400 °F, and wet stacks, where moisture content can be 30–40% at 200 °F.

The instrument system has been tested and approved for emissions monitoring at two incinerator sites that represent the extremes in stack types. Limits of detection for the 14 HAP metals are in the 0.1–5 g dscm$^{-1}$ range. QCs are automatically checked approximately every 8 h (in 24-h operation), with demonstrated calibration stability of >2 weeks. Calibration and QC checks are fully automated and executed at user-selectable intervals in this turnkey instrument system.

### Use of an automated liquid-handling system with a collision-cell ICP-MS for interference removal in elemental speciation

*Fergus Keenan, Thermo Elemental, 27 Forge Parkway, Franklin, MA 02038, USA*

The use of an integrated liquid-handling system for elemental speciation analysis will be described. The liquid-handling system consists of two switching valves, a pair of two-channel independently controlled variable-speed peristaltic pumps combined with a dual six-way pneumatic switching valve. The ICP-MS software operating under a Windows NT/2000 environment controls the system. Applications include microdilution techniques (on-line dilution, on-line addition of internal standards, on-line standard additions) continuous and segmented flow hydride generation, low-pressure chelation chromatography and rapid sample throughput applications.

Data will be presented for the analysis of chromium species by low-pressure solid-phase chelation (SPC) ICP-MS. Of the two inorganic forms of chromium, Cr(VI) is a known carcinogen, while Cr(III) is an essential element. Total chromium measurements, therefore, cannot be used to determine actual environmental impact due to the considerable difference in toxicity between the two elemental forms. Initial investigations using automated SPC with ICP-MS for chromium speciation have recently been reported [1]. Limiting factors of this ICP-MS method are the polyatomic species 52ARC and 53ARC, which can interfere with low-level detection of the two most abundant chromium isotopes. Results will be presented on the use of a collision-cell-equipped ICP-MS to remove the polyatomic argon carbide species for the analysis of chromium species.

1. Donais, M. K., Henry, R., and Rettberg, T, 1999, *Talanta*, **49**, 1045.

### Monitoring biospecific reactions by refractometry

*Thomas E. Ryan[1], Robert Atkinson[1], Christine Campagnolo[2] and Carl Batt[2], [1]Leica Microsystems, Inc., Buffalo, NY, [2]Cornell University, Ithaca, NY, USA*

This study compares sensitivity, specificity and response rates of critical angle and surface plasmon resonance (SPR) refractive index-based biosensors. A commercially available reflected light refractometer was used to study both critical angle and SPR responses. Glass slides (critical angle) and gold-coated glass slides (SPR) were optically coupled to the refractometer prism using a high-index coupling liquid. Binding of biomolecules at the slide surface results in a refractive index change due to high localised mass addition. Surface refractive index was monitored in real time during surface formation, immobilisation of biomolecules and interaction of complementary binding partners and controls to the immobilised molecule. A variety of chemistries were utilised to couple the proteins to the slide. SPR surfaces studied included streptavidin or neutravidin, a novel DTSSP derivatised streptavidin (neutravidin) surface and polystyrene on gold. Binding models studied by surface plasmon resonance include: (1) streptavidin: biotinylated goat anti-mouse IgG: mouse IgG; (2) streptavidin: biotinylated mouse IgG: goat anti-mouse IgG; (3) avidin: anti-avidin; and (4) mouse IgG: goat anti-mouse IgG.

The refractive index response to antigen, as measured by SPR, exhibited a high degree of specificity in comparison with control experiments using non-antigenic IgGs and BSA. The response was concentration dependent. Typical response plateaus were reached within 30 min at high levels of antigen ($50\,\mu g\,ml^{-1}$).

The refractive index response levels after 10 min of antigen exposure ranged from $5 \times 10^{-3}$ refractive index units at $50\,\mu g\,ml^{-1}$ antigen to $10^{-3}$ refractive index units at $5\,\mu g\,ml^{-1}$ antigen.

The noise level was typically $\pm 1.5 \times 10^{-6}$ refractive index units. The instrument response to refractive index changes was linear as determined by measurements made on standard sucrose solutions. Surface chemistries for critical angle experiments include adsorbed protein by ionic interactions, amine coupling to aldehyde terminal silanes, neutravidin-conjugated proteins on biotinylated glass, biotinylated proteins to streptavidin-coated glass and carboxymethyl dextran. Antigen/antibody binding, as measured by a critical angle refractometer, showed a high degree of specificity, a linear response to varying concentrations of antigen and a strong dependence on the type of surface chemistry employed for immobilisation. Antibodies immobilised directly to glass were very susceptible to loss of binding activity during attempts to dissociate bound antigen. Response levels monitored by critical angle were generally much lower than responses determined by surface plasmon resonance. An exception to this was the carboxymethyl dextran surface that produced an enhanced critical angle response and protected antibodies from loss of binding activity during surface regeneration. Existing theoretical models of the measured critical angle response are inconsistent with the results. Work is currently underway to understand and characterise better total internal reflection and light-scattering effects from thin biolayers.

### Speciation of inorganic selenium and seleno-amino acids by HPLC-hydride generation-atomic fluorescence spectrometry

*W. T. Corns[1], P. B. Stockwell[1], I. Ipolyi[2] and P. Fodor[2], [1]P. S. Analytical Ltd, Arthur House, Crayfields Industrial Estate, Main Road, Orpington BR5 3HP, UK, [2]Department of Applied Chemistry, Szent Istvan University, Budapest, Hungary*

The importance of selenium as an essential nutrient has long been recognized. More recently, it has been shown that biological samples often contain selenium as analogues of thio-amino acids. Seleno-amino acids are known to play an important role for enzymatic systems and have also been reported to show a protective effect against cancer.

Numerous countries have selenium dietary levels well below the WHO guidelines and this has resulted in vitamin supplements enriched with selenium becoming commercially available. These are often produced from selenium yeasts. However, little is known about the fate and metabolic pathways of these species.

Instrumentation based on HPLC-hydride generation-atomic fluorescence spectrometry will be described for the speciation of inorganic selenium and seleno-amino acids. The system was optimized using univariant optimization to achieve optimal separation, hydride generation yield and detector performance. It can couple almost all standard HPLC systems to a PS Analytical Millennium Excalibur, which still retains its ability to analyse samples for total selenium.

Studies to date have shown that the seleno-amino acids (selenocystine, selenomethionine and selenoethionine) can be converted to hydride species, thus eliminating the need for complicated on-line digestion routines. The analytical performance will be presented along with results for a wide range of food samples enriched with selenium supplements.

Detection levels comparable or better than other, more expensive techniques are obtained, which makes the choice of this equipment for the validation of data on the selenium levels in a wide range of samples extremely cost-effective.

### Integrated on-line analyser system for carbon dioxide, incorporating a new rack-mounted GC

*David J. Burges, Andrew H. Ditchman, David P. Miller and Martin J. C. Smith, Thermo ONIX, Viking Way, Bar Hill, Cambridge CB3 8EL, UK*

Current product purity specifications for food/beverage-grade carbon dioxide include maximum tolerable concentrations for a wide range of potential contaminants. Integrated analyser systems for $CO_2$ normally incorporate a number of dedicated monitors, such as oxygen, moisture and total sulphur analysers. However, many of the potential contaminants can only be measured practically by gas chromatography.

A new rack-mounted Unicam GC has been developed that can be readily integrated into on-line $CO_2$-monitoring systems, while maintaining the high performance and flexibility of the best laboratory gas chromatographs. The GC is fitted with the pulsed discharge ionization detector (PDID) for the analysis of trace impurities down to ppb levels. It can also be equipped with other standard detectors such as TCD, FID and FPD, and with multiple valves for multidimensional chromatography.

An integrated analyser system will be described with facilities for automatic stream selection and automatic calibration. The system incorporates the new GC with aluminium oxide moisture analysers and pulsed UV fluorescence analysers to determine total sulphur and total aromatics. The system includes unique ProLink software for comprehensive system control and reporting. ProLink provides automatic analysis of continuous and on-demand sample streams, analyser control including calibration sequences, and continuously up-dated display of latest results, analyser status and alarm conditions.

Chromatograms, results and data obtained for the analysis of beverage-grade $CO_2$ will be presented.

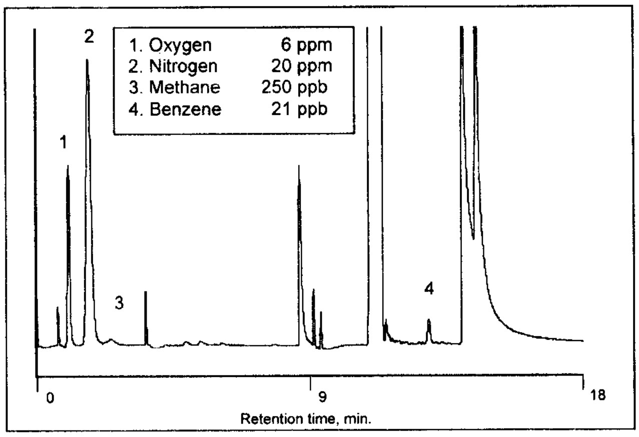

*Chromatogram of a beverage-grade $CO_2$ standard blend.*

### Determination of arsenite in food using hydride-generation spectrophotometry

*Wang Bingwu, Ren Hongxlng, Hu Tao and Wu Mingjia, Changchun Institute of Applied Chemistry, Chinese Academy of Sciences, Changchun, Jilin 130022, PR China*

Speciation of elements in biological, environmental and toxicological sciences is important because different species have different bioactivity, metabolism and toxicity. Inorganic arsenic exhibits two forms: arsenite and arsenate. The former is more toxic than the latter.

We proposed a simple, rapid and reliable method to detect and analyse arsenite's content. The detection of trace arsenic using spectrophotometric method and silver salt was modified. The method limit of mass detection is 0.2 g. The accuracy and precision of this technique and its application in the analysis of a soybean product will be discussed.

The overall sampling and analysing procedures were carefully designed and optimized for the best arsenite's detection. To keep the sample in its original oxidation state, selective analysis of arsenite were perform for samples acidified with 2 M HCl at $<100\,^{\circ}C$.

Unlike the conventional method, the reduction of As(III) to $AsH_3$ takes place in phosphate matrix at pH 5–6 using potassium borohydride tablets as reductant. Potassium borohydride tablets were made by mixing dry potassium borohydride and sodium chlorite powders in a ratio of 1:5 (weight) and pressing them to tablets at weight of $1.5 \pm 0.1$ g. This method greatly simplifies the procedure and is ideal for routine analysis. Some additional experimental precautions will also be discussed.

The $AsH_3$ is trapped in an adsorption solution, which is a mixture of acetic acid, silver nitrite, nitrite acid, polyvinyl alcohol and alcohol.

The stability of the adsorption solution was improved by adding acetic acid. By using alcohol instead of chloroform, we also minimized the healthy danger involved in the measurement.

### Determination of trace elements in forensic bullet samples by plasma source mass spectrometry—1. Removal of lead by flow-injection solid-phase extraction

*Emily R. Yourd[1], Julian F. Tyson[1] and Robert D. Koons[2], [1]Department of Chemistry, University of Massachusetts, Amherst, MA 01003-9336, [2]FBI Academy, FSRTC, Quantico, VA 22135, USA*

Solid-phase extraction materials have been examined for the selective removal of lead from solutions of bullet samples.

Chromosorb 102, silica-immobilised 8-hydroxyquinoline, *o*-vanillinthiosemicarbazone immobilised on Amberlite XAD-2, dicyclohexano-18-crown-6 in decanol immobilised on Amberlite XAD-2, and Pb-Spec (Eichrom, Inc.) were compared on the basis of capacity as well as specificity for lead over the target trace elements. The extractants were each packed into a column and incorporated into a flow-injection manifold.

Acid-digested bullet samples were injected into the system and the interfering lead matrix was retained while the analytes passed through the column to the detector. The most successful matrix removal was accomplished with the Pb-Spec resin, which removed 99.5% of the lead from a 3% $HNO_3$ carrier solution. The lead was quantitatively eluted with a solution of 0.1 M ammonium citrate, regenerating the column. The analytes Ag, As, Bi, Cd, Cu, Sb and Sn were determined by ICP-MS, and the method was validated by the analysis of NIST SRMs 2415 (battery lead), 2416 (bullet lead) and 2417 (lead-base alloy). The possibility of extending the suite of analytes to include additional elements has been evaluated.

### Elemental profiling of trace evidentiary materials by laser ablation inductively coupled plasma mass spectrometry (LA-ICP-MS)

*Elzbieta (Ela) Bakowska[1], Joann Buscaglia[2], Robert D. Koons[2] and Stephen Shuttleworth[3], [1]Agilent Technologies, ASMC, 2850 Centerville Road, Wilmington, DE 19808, [2]FBI, Forensic Science Research Unit, FBI Academy, Quantico, VA 22135, [3]New Wave Research, 178 Riverside Avenue, Warwick, RI 02889, USA*

The measurement of trace elemental profiles may be used for classification and discrimination of many types of mass-produced materials. The main challenges of traditional elemental profiling materials are: small sample size, the destructive nature of some methods of analysis, sample preparation and contamination. Direct analysis of solid samples is a preferred method for forensic materials.

Laser ablation (LA) practically requires no sample preparation and uses so small quantities of the sample that the method could be labeled as non-consumptive. The sample-to-sample analysis time takes only several minutes. Since inductively coupled plasma mass spectrometry (ICP-MS) offers exceptional detection limits, the sample size is not an issue. One of the main limitations of the use of a standard ICP-MS instrumentation (without laser ablation) for forensic applications is the sample preparation. The traditional way of introducing a sample into ICP-MS is by aspirating the sample solution. Solid samples require additional steps in converting them into a liquid. Those extra procedures are potential sources of contamination, loss of the analytes, require more time, and the use of hazardous chemicals. Also, during the sample preparation, the sample is consumed and/or altered from the original form. The main disadvantages of the LA-ICP-MS are the lack of calibration standards for most of the matrices, and the manual mode of operation (no autosamplers are available).

Those disadvantages are minor for the direct comparisons between crime scene and suspect-associated materials. For several sample matrices, the solid standards are available. The examples of application of LA-ICP-MS to the elemental analysis of ink on paper, glass fragments, hair, synthetic fibers, paint chips and sheet polymers will be presented.

### New approach to unattended automated HPLC method development

*T. Jupille*[1], *L. R. Snyder*[1], *J. W. Dolan*[1], *R. Lee-Berman*[1], *M. Swartz*[1] *and K. Wlpprecht*[2], [1]*LC Resources, Inc., 2930 Camino Diablo, Walnut Creek, CA 94596,* [2]*Waters Associates, 34 Maple Street, Milford, MA 01757, USA*

Automated unattended development of HPLC separations has been a 'holy grail' for chromatographers for over a decade and a half. Previous approaches have focused on the optimisation of a single parameter (e.g. the ratio of different organic solvents in a reversed-phase separation) under restricted conditions (e.g. iso-eluotropic separations). They have found limited acceptance because even the 'optimised' conditions often fail to provide adequate separation. As a consequence, a great deal of sample, solvent and instrument time can be expended with little useful information to be gained in return. Clearly, a new approach to the problem is required.

The present approach combines computer modeling of chromatography behavior with sophisticated instrument control to identify automatically adequate separation conditions over a broad range of separation parameters. It begins by allowing the user to specify goals for the analysis and key information about the nature of the sample. These form the basis for an initial screening strategy to identify which of four chromatographic variables (solvent strength, temperature, solvent type, pH) are most likely to yield an adequate separation given appropriate manipulation. This strategy is automatically implemented by the chromatography hardware. The resulting data are used by the system to identify and verify specific sets of conditions that yield adequate results.

Preliminary work with published retention data suggests that this approach is capable of reliably identifying adequate conditions (critical resolution >2) for moderately complex samples (up to a dozen components) without user intervention. This paper will discuss the underlying models and strategies and demonstrate application to real separation problems.

### Real-time expert system for automation of HPLC method development

*S. Galushko, V. Tanchuk and I. Shishkina, Im Wiesengrund 49-b, 64367 Muehltal, Germany*

The objective was to develop a real-time Expert System, which can perform unattended HPLC method development. To automate the intelligent behaviour of a chromatographer, an approach was applied that involves combination of artificial intelligence (AI) methods with mathematical procedures and Microsoft Automation technology. The System controls on-line HPLC software and hardware, provides dynamic data exchange, models chromatographic behaviour of compounds as a function of chromatographic variables (structure parameters, RP column properties, solvent concentration, gradient conditions, temperature) and searches for optimal conditions. The AI module of the system models actions of a chromatographer to evaluate results, to solve problems and to make decisions. The behaviour of the system is based on the build-in knowledge and its own experience. Therefore, the system is really autonomous and performs unattended method development overnight. The system separates mixtures of target compounds and can search for optimal concentration of an organic solvent, optimal linear and multisegment gradient profiles and temperature for reversed-phase HPLC. The system can try several columns and solvents and proposes alternative conditions for the separation.

The system is built in ChromSword-Auto HPLC method development software (Merck KGaA, Darmstadt, Germany) and controls HPLC software and hardware (Merck-Hitachi LaChrom 2000). It has been successfully tested for the fully automated developments of HPLC methods for the separation of various mixtures (three to 20 compounds) from the pharmaceutical, chemical industry and environment.

### Automated method development of HPLC methods for complex pharmaceutical assays

*Michael E. Swartz*[1], *P. A. Fowler*[1], *L. R. Snyder*[2], *J. W. Dolan*[2], *R. Lee-Berman*[2] *and T. Jupille*[2], [1]*Waters Corp., 34 Maple Street, Department TG, Milford, MA 0175,* [2]*LC Resources, Inc., 2930 Camino Diablo, Suite 110, Walnut Creek, CA 94596, USA*

HPLC method development of complex pharmaceutical assays such as those used for related compounds or stability testing is a time-consuming process. While trial and error approaches are still pursued, many researchers prefer a more efficient path and utilise chromatographic modeling software that relies on theory to decrease the time and resources required. However, while automated HPLC systems exist to run the methods, there is a disconnect between the chromatographic software and the modeling software. This situation results in a manual process that requires operator intervention for interpretation and implementation.

This presentation will summarise a new approach to HPLC method development that automates the entire process from method requirements/definition to method implementation.

This holistic automated method development approach consists of both software and hardware operated by an iterative decision engine driven by a graphical user interface.

Following input of basic separation requirements, starting conditions are identified and run, evaluated by the modeling software, optimised and automatically implemented by the chromatographic system. The iterative process is repeated until the separation goals are achieved. The result is an automated method development system capable of unattended operation that increases the throughput and efficiency of the method development laboratory. We will demonstrate the use of this system for the development of complex pharmaceutical assays that take advantage of the selectivity afforded by high pH mobile phases and columns specifically designed for that purpose, as well as additional software tools available for method development.

### Intelligent optimisation of HPLC separations based on chemical structures: benefiting from a unified knowledge base

*Robert S. Dewitte, Michael McBrien and Eduard Kolovanov, Advanced Chemistry Development, 702-90 Adelaide W., Toronto, Ontario M5H 3V9, Canada*

By combining prior knowledge with chemical insight, determining robust HPLC separation conditions can be streamlined. Advanced chemistry development provides software tools for both chromatography method development and the prediction of molecular physical properties. Measurements themselves can be taken for many purposes: the accuracy and applicability of both suites of tools can be improved by contributing experimental results generated within your laboratory, at the laboratory of a collaborator or by a group examining compounds for completely different purposes. This 'user training' is cooperative in the sense that experimental results used to enhance a computed molecular property can enhance the reliability of a chromatographic simulation and vice versa. By way of example, this talk will focus on the benefits of establishing a unified company knowledge base through ACD products: synergistic enhancement of accuracy and applicability.

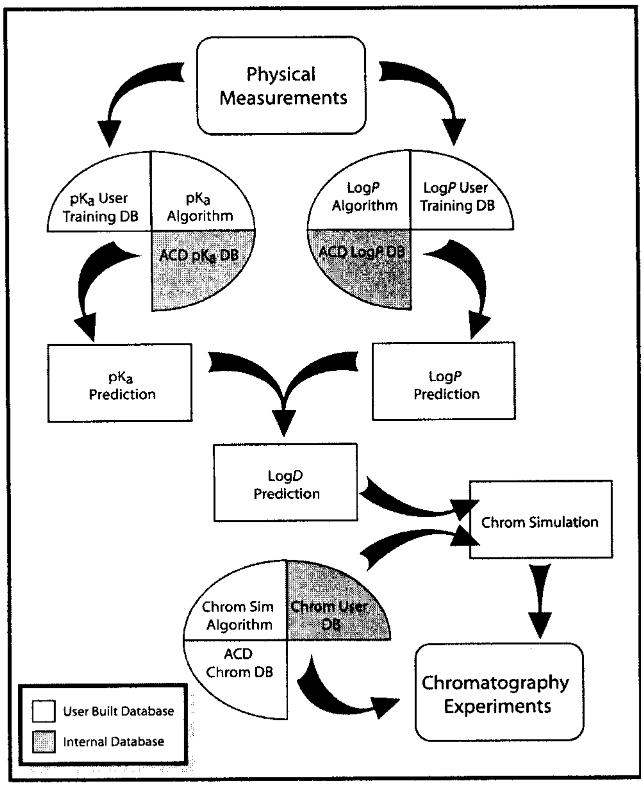

### New computer-control LC-CLND: automatic quantitation for open-access laboratory

*Jean-François A. Borny*

In a perfect world, machines will do the menial task and chemists will do research & development, invent new technology and discover new wonder drugs. The HPLC-CLND is a significant and important detector in pharma research, yet the menial task of setting flows, putting the detector in standby, changing gain setting are still left to the chemist—until now.

The new computer-controlled HPLC-CLND allows the chemist simply to set his sample in the queue, type in the password or method, and walk off, knowing the computer will set the right method for all detectors, analyse the samples, rerun any over-range samples at the appropriate gain, and set the detectors in standby mode once the analysis are finished. This presentation will describe the ultimate, automated, open-access laboratory.

### High-throughput purification of combinatorial and focused libraries with on-line MS analysis

*Reinhold Spatz[1], Artur Meisner[1] and Kisaburo Deguchi[2], [1]Merck KGaA, SLP, 64271 Darmstadt, Germany, [2]Hitachi, Instr. Div., 882 Hitachinaka, Japan*

Methods of combinatorial chemistry or parallel synthesis are used to accelerate the lead finding and lead optimization process in drug and chemical development. The big number of synthesis products generated by combinatorial chemistry has to be purified and analysed before screening of their biological activities. To handle these many samples, the high throughout purifier mass spectroscopy system (HTP-MS) was developed, which can purify synthesis products while running four purification columns in parallel.

The target molecular weights for the desired synthesis products are analysed on-line in parallel with a commercial ion-trap mass spectrometer capable of discriminating information from the parallel purification process. The MS also can be used to trigger the fraction collection for the parallel purification process based on the preset target mass. A PC software (HTPM-MS) controls hardware and processes all data from the four-channel HPLC and from the ion-trap MS. The data may be analysed on the desktop or qualified in terms of yield/purity and target mass used to trigger fraction collection.

Working principles and related hardware and software will be presented as well as a big variety of application results, which were achieved in the lead finding process of pharmaceutical drug development with synthesis and natural products.

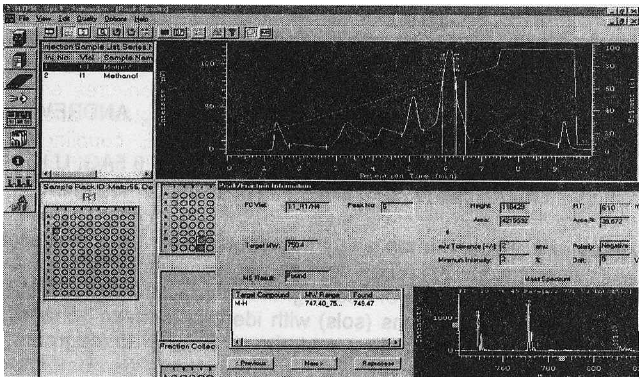

*Mass triggered purification of a component in a complex synthesis mixture. The peak fraction information window confirms the target mass by a mass spectrum of the collected fraction.*

**Advances in the application of array detectors for improved chemical analysis**

*M. Bonner Denton, Department of Chemistry, University of Arizona, Tucson, AZ 85721, USA*

Over the last 15 years, focal plane array detectors have brought an amazing revolution to analytical spectroscopy. These devices have dramatically changed atomic emission spectroscopy by allowing simultaneous observation of the entire spectral region or selected subregions, providing increased sensitivity, requisite dynamic range and greatly improved background correction. Focal plane arrays have also vastly increased sensitivity in many other areas of low-light spectroscopy, including fluorescence, phosphorescence and Raman.

Recent advances in utilising these detectors for spectroscopy and spectroscopic imaging will be presented. Studies of the inductively coupled plasma (ICP) in the vacuum UV have yielded improved analysis of non-metals, as well as a host of highly promising, previously unobserved lines for many metals. Results of these investigations will be presented.

The use of Raman spectroscopy as a rapid method for identification of gems and minerals will be discussed, and ongoing progress in the generation of a gem and mineral database will be presented.

The impact of recent technological breakthroughs in array detectors, optical components and geometries of optical systems employing both dispersive and imaging optics will be contrasted with more conventional approaches.

Current trends will be assessed and utilised to predict future application of improved focal plane arrays to many areas of modern chemical analysis, ranging from X-ray to IR and even to mass spectrometry.

**Key role of scientific databases in the next millennium**

*F. Jack Smith, School of Computer Science, Queen's University of Belfast, Belfast BT7 1NN, UK*

Scientific research and discovery has always been based on data: without astronomic data, Isaac Newton would not have discovered his theory of gravitation. Good engineering design has also been based on data: without data on materials, plasmas and astronomy, man would not have walked on the Moon. However, just one scientific generation ago, the data consisted of a limited number of facts and quantities found in textbooks, books or journals. Today with the advent of computers, particularly personal computers, which can store gigabytes of data, and the Internet, which makes the data instantly available across the globe, the nature and scope of much scientific and engineering data, and in consequence of much scientific research, has changed. Measurement technologies have improved in quality and quantity with measurement times reduced by orders of magnitude. Virtually every area of science—astronomy, chemistry, geosciences, physics, biology and medicine—is becoming model based, dependent on large bodies of data held in scientific databases. In the same way, modern engineering design depends on large scientific databases on the properties of materials.

All these advances point to an ever-increasing need for more data, better access to them and higher data quality, stored in large interrelated databases. Already thousands of databases are available, and the numbers grow weekly. These databases deliver a rich variety of scientific information including numeric data, images, maps, videos, expert systems and full text of research articles, abstracts and indexing information. Almost all of science and engineering in the coming millennium will depend on the correct and imaginative design and implementation of these scientific databases, all available on the Internet.

All this activity has naturally begun to spawn a lively body of literature, growing rapidly in volume, detail and breadth. However, it is still in its infancy. The study of these databases is itself a large subject covering the data themselves, the databases and their application.

Data science includes the study of the capture of data, their analysis, metadata, fast retrieval of data in a large database or across the Internet, archiving of data so that they are available for future generations, exchange of data and quality of data. Database research includes interfacing data, data mining to find unexpected data and data relationships, visualization of data in two and three dimensions including movement and management including intellectual property rights and other legal issues. Adding intelligence, still in its infancy, will be a growing subject with long-term benefits, which are hard to predict but may be most significant of all. So scientific databases will surely have a key roll in all science and engineering in the coming millennium. It follows that this vital subject will need quality journals to aid the study of all aspects of scientific data and databases, those described above and more, in a subject we might call 'Data Science'.

### Data quality versus data quantity

*John Rumble Jr, National Institute of Standards and Technology, 100 Bureau Drive, Gaithersburg, MD 20899-2310, USA*

It is a common assumption, though unproven, that the Internet era and information explosion have reduced the quality of scientific data, especially in databases. Regardless of whether this is true, it is timely to look at the issue of data quality in terms of scientific databases. First, we must differentiate between two types of scientific databases: compilations of reported results and compilations of critically evaluated data. The first merely gathers together measurements and calculational results as reported in the literature. The latter is a selection, and possibly adjustment, of all or some of the reported results, screened according to some set of criteria. The difference reflects the fact that a single measurement does not constitute a property. Indeed, properties are derived from many measurements and usually take many years to establish.

NIST has maintained its Standard Reference Data Program for several decades. In the course of this work, certain principles related to data evaluation have become apparent. Specifically, scientific data evaluation combines three perspectives: (1) the identification of all independent variables, control thereof and documentation of that control; (2) the adherence of the data to the known laws of nature; and (3) the comparison of the data to other measurements that purport to examine the same phenomena. The mixture of the three viewpoints depends on the maturity of the science and the status of previous data evaluation efforts. It is important to recognise that rarely does a body of repeated measurements exist that allows for statistical analysis that might be naively expected.

However, the quality of scientific databases depends on more than the quality of the data contained therein. Again, in its database program, the NIST has developed a number of principles related to testing the quality of databases themselves. The criteria are as follows.

- The correctness of the retrieval results, i.e. the correct data value is retrieved every time, and all data meeting the specified criteria are reported.
- The performance of the database, i.e. no crashes, easily to use, no ambiguity in specifying the property or substance.
- Consistency of performance, i.e. the database and searches work the same way every time. In addition, good scientific databases must recognise that users are accustomed to a variety of nomenclature systems, both for substances and properties. For the highest quality, a scientific database must not mislead users in terms of the completeness of results because the lack of synonym tables and translation of terms.

Emphasising the quality aspects of data and databases does not means that data quantity does not matter. Many times, important research leads or analytical results depend on access to complete though fragmentary information. Because science is such a non-linear process, data compilations without data evaluation still play an important role. The above points will be illustrated through examples from databases in analytical chemistry, structural chemistry, the fundamental physical constants and materials.

### Automated measurement of carbon and hydrogen content of refinery liquids using GC-AED

*Bruce D. Quimby[1] and David A. Grudoski[2], [1]Agilent Technologies, Wilmington, DE 19808-1610, [2]WeMeasureIt, Inc., 2342 Shattuck Avenue, #59, Berkeley, CA 94704-15177, USA*

Hydrogen plays an important role in the refining of petroleum. Knowledge of the carbon and hydrogen content of a given stream should be useful in selecting appropriate process parameters for maximum efficiency. Gas chromatography with atomic emission detection (GC/AED) can measure carbon and hydrogen simultaneously. By integrating the total area of the carbon and hydrogen chromatograms and comparing them with a standard, the relative amounts of carbon and hydrogen in the sample can be determined. If the chromatographic time axis is calibrated with hydrocarbons, the hydrogen content can be determined as a function of boiling point as well. The hydrogen response of the AED exhibits two problems that have previously limited the accuracy of the hydrogen measurement: relative to the carbon response, the hydrogen response tails and is non-linear.

This talk will describe a new method that addresses both problems. In the method, an EPC-controlled flow of methane is added into the detector make-up gas. The addition of a small percentage of methane into the make-up gas eliminates the tailing of hydrogen relative to carbon. The EPC-controlled methane addition is further used to provide automated calibration of the linearisation correction for hydrogen and the relative response factors for carbon and hydrogen. These calibrations are then used to calculate the total percentage carbon and hydrogen in samples. The carbon and hydrogen data can also be used to construct a chromatogram where the response is directly proportional to the amount of a compound and its degree of hydrogen deficiency. These 'aromatograms' are useful for representing, for example, aromatic compounds in the presence of saturates.

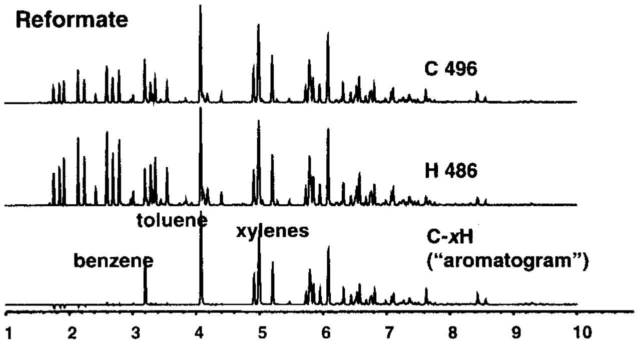

*Chromatograms of reformate.*

### Identification and quantification of pesticides in food matrices with minimal sample preparation using a GC/TOFMS

*Ritchard Parry, Evaldo de Armas, Jack Cochran and Nancy Myers, LECO Corp., 3000 Lakeview Avenue, Joseph, MI 49085, USA*

Organochloro pesticides (OP) are among the most widely used pesticides in agriculture. These types of pesticides are very effective, but at the same time they exhibit high toxicity. It is of particular interest to monitor the pesticide residue levels present in food materials especially when these food items are used in baby food formulations.

The analysis of food samples usually requires some type of extraction mechanism to collect and concentrate the pesticides of interest. In addition, the matrix components complicate the chromatography requiring long chromatographic times to ensure complete separation.

A time-of-flight mass spectrometer (TOFMS) offers several unique advantages for reducing analysis times. The unique degree of spectral continuity across a chromatographic peak provided by TOFIMS systems has allowed the development of several revolutionary software algorithms that allow deconvolution of peaks to be performed, effectively resolving the mixed mass spectra of the co-elution into accurate individual mass spectra for each analyte, including the accurate distribution of signal from masses shared by several components in the co-elution.

The potential benefit of these unique features were evaluated by the rapid screening of organochloro pesticides by GC/TOFMS.

In this presentation, examples will be given on the use of a TOFMS system coupled to a gas chromatograph for the analysis of food matrices using fast chromatographic programs. Examples will be shown on automatic deconvolution and its use to resolve coelutions minimising the contribution of the matrix components.

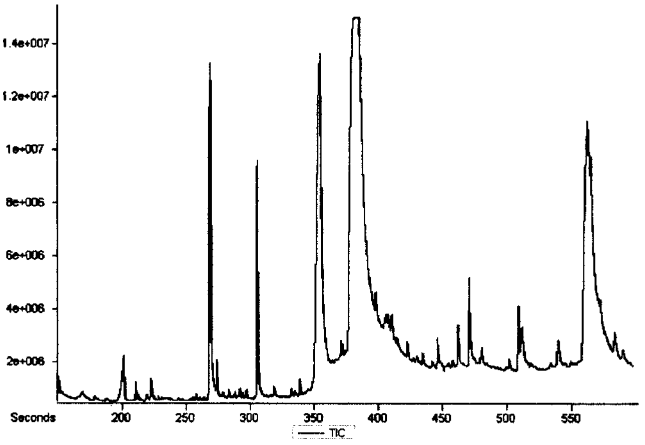

*Chromatogram of an apple sauce extract.*

### Development and application of a preparative-scale supercritical fluid chromatography system for high-throughput purification

*Jill Hochlowski, Philip Searle, Geewananda Gunawardana, Thomas Sowin, Jeff Pan, Jeff Olson and Jonathan Trumbull, Medicinal Chemistry Technologies, Abbott Laboratories, Abbott Park, IL 60064, USA*

A preparative-scale supercritical fluid chromatography (SFC) system has been added to the sample purification capabilities of the high-throughput purification (HTP) group of Abbott Laboratories Pharmaceutical Products Drug Discovery.

This system was modified from a commercially available instrument to accommodate automated sample handling, provide robust peak detection and collect an arbitrary number of fractions per sample with higher compound recovery than commercially available packages. An informatics package was developed to integrate this technique into the currently existing purification system, such that SFC and HPLC were seamless, interchangeable purification options.

A wide range of chromatographic methods was investigated through which a series of standard protocols were developed applicable to processing pharmaceutical samples. This talk will detail our experience to date with this system for the purification of samples generated by both medicinal and combinatorial chemists.

### On-line extraction with capillary HPLC/MS for fast, sensitive drug analysis

*Cozette M. Cuppett and Steven A. Cohen, Waters Corp., Milford, MA 01757, USA*

As pharmaceutical development is being increasingly directed at high-potency drugs, it is necessary to utilise highly sensitive *separation* methods for analysis. When performing pharmacokinetic studies on these potent drugs, samples are mass limited and can require a considerable amount sample clean-up before injection. Capillary liquid chromatography with on-line extraction has the potential to address the sensitivity, mass limitation and sample clean-up issues related to the analysis of these new pharmaceuticals.

A fully automated capillary LC/MS system with column switching capabilities was used to isolate analytes from plasma using a trapping column, followed by elution and separation of the analytes on an analytical column. Silica-based C,8 and polymeric stationary phases were used in the trapping columns. Short capillary columns ($0.32 \times 50$ mm) were employed with fast, steep gradients (5–95% organic in 2–4 min) to elute the samples for MS detection. The total analysis time for samples was $\leq 7$ min, and sample volumes up to 20 μl were analysed. MS detection allowed for quantitation of certain molecules, such as propranolol, down to the low picogram level.

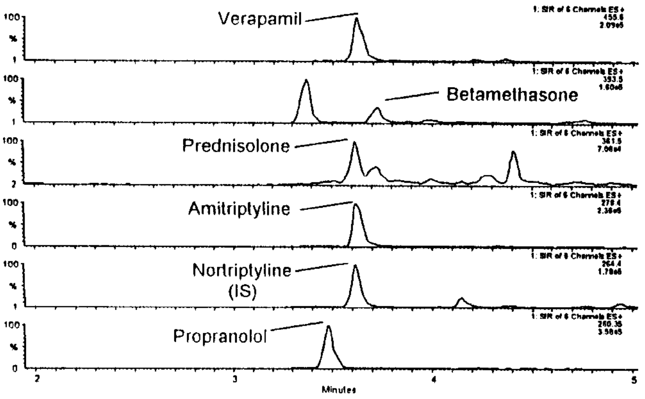

*Single-ion recording chromatograms of a 1 µl injection of plasma spiked with standards (50 pg of each component on column). Sample was loaded onto an extraction column, separated on an analytical column and then detected with a single quadrupole mass spectrometer.*

### Wireless local area network computing devices expand the LIMS environment

*Paul A. Fjeldsted and Gary Gostecnik, Analytical Automation Specialist, 11723 Sunbelt Court, Baton Rouge, LA 70809, USA*

The traditional role of a LIMS is collecting data in the laboratory, and then generating reports for the organisation's use. With the advent of easy to use LIMS interfaces and wireless local area networking, the value of the LIMS can be enhanced by collecting data at the source and providing information where it is needed. Wireless technology can improve the timeliness of the data and its availability. Examples of applications where this can benefit the users of the LIMS are: sample collection, data collection and real time access.

When used for sample collection, the LIMS-generated sample ID can be assigned to the sample at the beginning of the sample's life and thereby reduce sample identification errors. This eliminates the need to use database replication or to merge off-line records.

When used for data collection the results are immediately available to rest of the LIMS users. Improved timeliness of data reduces the amount of resampling and increases the quality by more accurately associating a result with conditions of a process.

Using wireless local area networking (LAN) reduces complexity in LIMS programming. The need for off-line databases, disconnected applications, and multiple databases are eliminated. This makes it easier for the application programmer to focus on adding value to the application instead of spending time on record management.

A wireless LAN is deployed using a wireless network interface card (NIC) and one or more access points. More than one access point is installed when the area covered is greater than can be covered by a single access point. When multiple access points are installed, the mobile user can transparently move throughout the site with wireless connectivity.

Wireless LAN performance range in speed from 1 to 11 Mbp. The application designers must take this into account as well as physical characteristics of the device the user will be using. At wireless LAN speeds, and with their propensity for interference, the type of network access is critical. The problems and advantages of using file access databases, client–server databases and thin-client access will be discussed.

The form factor of the client device dictates the type of user interface that is viable. Many devices well suited for wireless use are handheld, using pen input instead of a keyboard. The application designer must take this into account as well as the screen size available to view the application. The advantages of different devices will be discussed.

### Delivering LIMS via the application service provider (ASP) model

*Tony Johnson and Kevin Smith, Thermo LabSystems, 1 St George's Court, Hanover Business Park, Altrincham WA14 5TP, UK*

The ASP model of delivering applications over the Internet as a rented service is receiving much press attention and is firmly established as the current 'new' thing in IT.

In the brave new ASP world, customers no longer buy software applications as a capital purchase and neither do they physically manage the applications on their own premises. Instead, applications are rented for a contracted period on a per user per month basis. The applications themselves are hosted on a third-party site with system maintenance, backup and recovery, etc. being provided by a third party.

The nirvana promised by ASP is a potent mixture of allowing business to focus on their core competencies, removing capital expenditure issues and reducing the need for customers to provide their own IT services and infrastructure.

This paper explores the reality of ASP, gives an overview of how the model works, describes some of the problems that can be encountered and the benefits it can deliver to customers.

### Flexible laboratory automation

*John Dickson, ThermoLabSystems Pathfinder, 1 St George's Court, Altrincham, Manchester WA14 5TP, UK*

Automation in any context always leads to loss of flexibility. This is particularly true for laboratory automation solutions involving hardware. However, it also applies to software automation solutions, e.g. LIMS.

Inflexibility is unavoidable and should be evaluated as a necessary cost during automation system cost–benefit studies: it is easy to overlook this cost. One strategy for minimizing this cost is to specify flexible automation systems. This strategy is fraught with danger because it leads to complex systems that are difficult to implement, validate and maintain. An alternative strategy is to implement simple, inflexible automation solutions using short project life cycles. It then becomes realistic to plan for regular upgrade or replacement as the business needs change. The costs and benefits associated with both each strategy will be examined together with practical examples taken from both laboratory robotics and LIMS. This presentation will be of interest to anybody involved in the design or maintenance of LIMS and other laboratory automation solutions.

### How to migrate a corporate LIMS system

*Miguel A. Fernandez, ThermoLabSystems, Av. Valdelaparra 27, Alcobendas, Madrid, E-28750, Spain*

LIMS systems can become eventually obsolete due to functional or technological obsolescence both in the LIMS itself or in surrounding platforms, company merges or simply by an evolution of the requirements unaccompanied by a similar evolution of the software in use. In a dynamic world, which was good once has not to be good forever. When the time to change has arrived, a corporation cannot afford to hide away and continue to use the existing LIMS or to undertake the change in an uncontrolled way. Either way is a potential source of chaos and loss of quality in the work of the laboratory.

There are technical issues, related to the new data structures, to the new hardware and software platforms used, and to the migration of valuable historical data. There are also functional issues regarding the change in procedures, and human issues, as people are not always ready to modify the way they have been working for years. The aim is to replace a piece in the corporate schema with no impact for the rest of the corporation systems, sometimes in critical environments, without affecting the service for a minute, and without a drop in quality.

The paper will present a structured approach to this process, in which all tasks are defined and scheduled for success in an otherwise difficult evolution.

### Laser-induced breakdown spectroscopy for automated metal analysis

*H. Falk and P. Wintjens, Spectro A. I. GmbH & Co., KG, Kastellstrale 31–35, 47546 Kalkar, Germany*

Laser induced breakdown spectroscopy (LIBS) can increase the speed and reliability of the automated analysis of metals if all relevant elements including non-metals such as gases and carbon can be analysed. With the intention to provide the required solutions to the steel-making industry and based on the wide expertize acquired in this field with spark spectrometry, a laser spectrometer has been developed that is suited for industrial applications. Based on the use of a Q-switched Nd:YAG laser with 50 Hz repetition frequency operating on the second harmonic at 532 nm, a gas-tight ablation/excitation stand, translation and rotation possibility for the beam-focusing unit, and the use of an adapted light collection and detection unit, the possibility to carry out element analysis of all elements of interest (120–800 nm) in the steel industry has been investigated.

In contrast to the spark OES with LIBS, the interaction area on the sample surface can be chosen from 50 μm to 1 mm. If in the industrial application speed is the major issue and not the investigation of inclusions, a larger focus diameter of >0.6 mm allows for best precision.

With the above described instrumentation, all elements relevant for the automated steel analysis could be determined with detection limits similar to those known from spark OES which applies also to the calibration graphs. To remove surface contaminations including oxides, 50 laser shots are sufficient. While applying the laser instead of spark OES, the non-homogeneities of the samples are of higher impact on the precision which has to be compensated for by averaging over a larger number of measuring points on the surface.

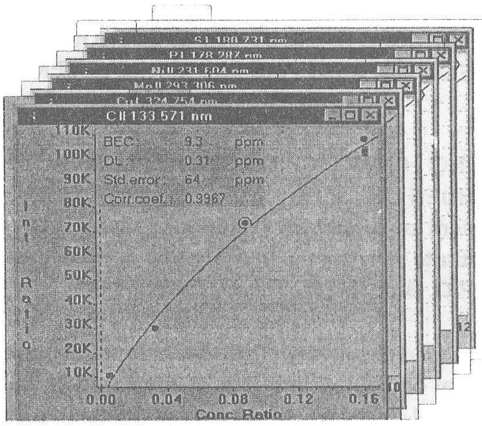

*Example of a calibration graph with LIBS for low alloy steel samples.*

### Integrated LC/MS chromatography information management system

*William C. Schnute, Dionex Corp., 1228 Titan Way, Sunnyvale, CA 94088, USA*

Information management is one of the most important issues confronting us today. Regulations and customer demands push instrument and data system developers to provide ever-greater volumes of information, and the automating of instrumentation turns out data faster than ever before. The implication for data intense 'hyphenated techniques' such as LC or IC mass spectrometry is that there must be development of better techniques for the access and interpretation of the data generated, converting it into usable information for the researcher.

For CHROMELEON-MS™, the application of mass spectrometric detectors to the various analytical methodologies used in liquid chromatography, and now ion chromatography, is addressed from the chromatographer's point of view. The data system can simultaneously process and displays data from LC detectors including UV, PDA, conductivity, fluorescence and a mass spectrometer in a format familiar to chromatographers. Display functions show information for each chromatographic peak, including simultaneous PDA and mass spectra. All detectors in the sample stream can be set with independent displays, both for intensity and for offsets. This gives multiple detector input for determining sample integrity and peak purity. The data system is a client/

server application that provides easy scale-up to remote monitoring and control of multiple instruments, and single-point access to any station on the network.

For mass spectral display, scans are displayed in real time, while additional information from extracted ion current profiles and SIM can be displayed for post-acquisition processing for both qualitative and quantitative applications. Display parameters are graphically selected for background subtraction, and results can be displayed by the use of scan filters for selecting the range and combinations of mass traces.

The reporting engine is based on embedded spreadsheet functionality, conforming to widespread spreadsheet convention, including formula expressions and charting of all available parameters and data. A complete set of GLP compliance tools is fully integrated into the software for security, validation and audit trail functionality. The system provides a complete set of features to help laboratories comply with 21 CFR 11, including features that facilitate the use of electronic signatures.

By the use of a unique combination of graphical displays, data formats and instrument control features, the management of the full range of information generated from chromatography/mass spectrometry is now available to the analyst and researcher.

### ASAP-MS: an automated software-structure analysis program for mass spectrometry

*Mike S. Lee[1], Antony Williams[2] and Vitaly Lashin[3], [1]Milestone Development Services, PO Box 178, Newtown, PA 18940-0178, USA, [2]Advanced Chemistry Development, 90 Adelaide Street West, Suite 702, Toronto, ON M5H 3V9, Canada, [3]ACD, Inc., ul. Akademika Bakuleva, 6, Str. 1, Moscow 117513, Russia*

The rapid growth of mass spectrometry (MS)-based software applications has been fueled by the unprecedented need to provide information necessary for decision-making [1]. Shorter timelines and a significantly greater number of samples has resulted in a tremendous focus on streamlined approaches that provide scientists, managers and executives the capability readily to obtain or even request the necessary information that leads to accelerated product development.

The generation of analytical data using roboticised high-throughput hardware has produced a bottleneck since data can be generated faster than it can be analysed. Thus, the recent emphasis on MS analysis strategies has been on producing information appropriate for decision. With decreasing costs and reduced footprints for instrumentation, more intuitive mass spectrometry software interfaces for non-specialists in an open-access laboratory environment is now a requisite in the industrial laboratory.

It is clear that there are distinct differences between the applications of mass spectrometry made available to non-specialists and that performed by the specialist. Whether the application provides data for synthetic chemists or expert spectroscopists, there is a need for a desktop MS processing, databasing and visualisation applications within a toolkit motif. In particular, the connectivity of such tools to an integrated molecular structure-handling capability including molecular fragmentation and structure searching is essential for today's structure-based directives [2]. This presentation will provide an overview of a recent approach taken to address these needs. Examples of a structure management system will be discussed with regard to specific MS applications and relationships with other analytical instrumentation.

1. Lee, M. S., and Kerns, E. H., 1999, *Mass Spectrom. Rev.*, **18**, 187.
2. Williams, A., Lee, M. S., and Lashin, V., 2000, An integrated desktop mass spectrometry processing and molecular structure manage-ment system. Advanced Chemistry Development Application Note, Toronto.

### Process monitoring of polymer composites during manufacture by FT-Raman spectroscopy

*Stuart Farquharson[1], Wayne Smith[1], Jennifer Rose[2], Montgomery Shaw[2], Elias Rigas[3] and Dana Granville[3], [1]Real-Time Analysers, 87 Church Street, East Hartford, CT 06108, [2]Institute of Material Science, University of Connecticut, Storrs, CT 06269, [3]US Army Research Laboratory, Aberdeen Proving Ground, MD 21005, USA*

The superior engineering properties of fiber reinforced polymer matrix composites, primarily the high strength-to-weight ratio, make them suitable to applications ranging from sporting goods to aircraft components (e.g. helicopter blades). Unfortunately, consistent fabrication of components with desired mechanical properties has proven difficult and has led to high production costs. This is largely due to the inability to monitor and control polymer cure, loosely defined as the process of polymer chain extension and cross-linking. To satisfy this need, we have developed an industrial 'hardened' FT-Raman instrument that employs fiberoptic probes for simple integration into polymer and composite manufacturing equipment. The instrument employs laser excitation at 1064 nm to avoid fluorescence interference, and an interferometer for wavelength separation with a single element detector to ensure wavelength accuracy and long-term stability. The instrument is immune to factory floor vibrations, shock and temperature drift, while performing continuous self-diagnostics, and providing real-time data collection and analysis. Real-time process

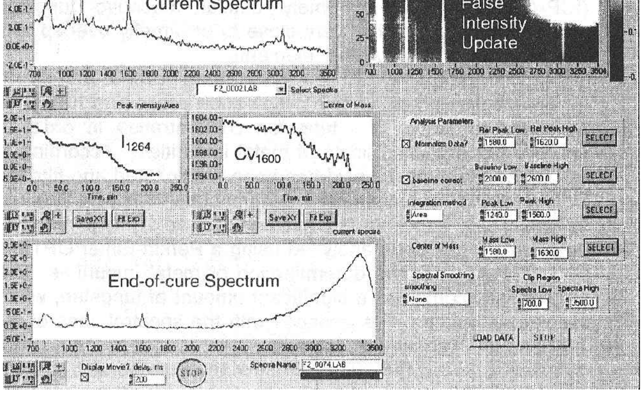

*FT-Raman analyser real-time spectra and data analysis display during cure of an epoxy resin composite within an industrial autoclave.*

Raman measurements will be presented for epoxy resins in a rheometer (simultaneous cure and viscosity) and epoxy resin and polyester composites in an autoclave (cure) (see the figure).

### Lab-on-a-Chip: application to water quality management

*John Proctor*[1] *and Ladun Odugbesan*[2], [1]*Yorkshire Water Services, 835 Main Street, Bridgeport, CT 06604,* [2]*BHC Co., 550 Huntington Street, Shelton, CT 06484, USA*

The analytical industry strives to provide its clients accurate results in the quickest possible time. Achieving this objective has global repercussions on process and product quality management. This paper describes the development and application of high-tech automated sensors capable of simultaneous multiparameter measurement. These sensors, developed from ceramic chips measuring 25 × 25 mm, replace time-consuming, costly and high maintenance laboratory testing. Although primarily targeting the water and wastewater industries, there is scope for application in other industries: food/beverage, environmental and pharmaceutical.

Two sensors tested in pilot studies include the CENSAR (pH, temperature, conductivity, DO, redox, free chlorine) and CLEARCENSE (color and turbidity) microchips. The system utilises thin/thick film printing on an alumina substrate with special inks to replicate conventional electrode systems and LED technology transferred from the telecomm arena. Advantage is taken of the fact that microdimensions are used and thus changes in the solution at the sample/sensor interface can be exploited.

Results of pilot studies show good correlation with grab samples and dynamic use as an early warning tool for investigating and avoiding potential negative events: pipe disturbances, pressure fluctuations or colored water calls to name a few. The trials have shown that the sensors can be reliably be deployed in pipeline systems at locations previously off limits to on-line water quality instrumentation. This has proved to be of immense aid in cases involving transitory excursions where the probability of capturing such an event by traditional sampling is extremely small. The modular nature of the deployment system has allowed several differing environments to be serviced—from raw water abstraction points to customer meters. In all instances where significant deviations from the normal have been recorded, causative factors have been identified facilitating remedial action.

### *In situ* real-time trace element monitoring in aquatic systems using a submersible voltammetric probe

*M.-L. Tercier-Waeber*[1], *J. Buffle*[1], *F. Confalonieri*[2], *G. Riccardi*[2], *A. Sina*[2], *F. Graziottin*[2], *G. C. Fiaccabrino*[3] *and M. Koudelka-Hep*[3], [1]*I(CABE), Department of Inorganic and Analytical Chemistry, University of Geneva, Sciences II, CH-1211 Geneva 4, Switzerland,* [2]*Idronaut Srl, Via Monte Amiata 10, I-20047 Brugherio (MI), Italy,* [3]*Institute of Microtechnology, University of Neuchatel, Jacquet-Droz CH-12007 Neuchatel, Switzerland*

There is a growing necessity to monitor continuously the levels of trace elements in the environment and in particular in aquatic systems. This stems from the fact that even present generally at very low concentrations ($10^{-12}$ to $10^{-7}$ M), these elements may present a severe hazard to the normal functioning of the aquatic ecosystem as they are not biodegradable but involved in various biogeochemical cycles and then distributed under different physicochemical forms (species). To understand and predict the role and the fate of these different metal species (in term of toxicity, bioavailability, bioaccumulation, coagulation, sedimentation, etc.), a new analytical instrumentation capable of performing *in situ*, real-time monitoring of specific forms, with minimum perturbation of the media, is required. The design of such tool is still a challenge for analytical chemists since techniques that combine high sensitivity, speciation capability, integrity of the samples and unattended operation are prerequisite. In addition, specific technical criteria such as miniaturization and robustness of the equipment, rapidity of data acquisition and transmission and low energy consumption are also important factors that have to be taken into account. A Voltammetric *In situ* Profiling System (VIP System) based on advanced microprocessor and telemetry technology and developed to allow real-time monitoring of trace elements in natural aquatic systems down to 500 m depth will be presented. This system combines high sensitivity, speciation capability, integrity of the samples with robustness, ease of handling and flexibility. The heart of the submersible voltammetric probe is a gel-integrated microsensor arrays specially developed to enable continuous, reproducible and reliable measurements of analytes in complex media without physical and *chemical* interferences of the test solution. The VIP system allows one to perform direct *in situ* measurement of the mobile fractions of Cu(II), Pb(II), Cd(II) and Zn(II) (ppt level) as well as Mn(II) and Fe(II) (ppb level). The characteristics of the probe and microsensor will be summarized. The VIP environmental features will be illustrated with examples of *in situ* applications in sea, lake and groundwater. Work underway to extend the application of the VIP System for direct *in situ* speciation of trace elements will also be presented.

### Total sulfur in combustion fuel utilising on-line process gas chromatography

*Ulrich Gokeler*[1], *Friedhelm Mueller*[2] *and Udo Offermanns*[2], [1]*Siemens Applied Automation, 7101 Hollister Road, Houston, TX 77040, USA,* [2]*Siemens AG, 76181 Karlsruhe, Germany*

Increasingly stringent environmental regulations will require the reduction of total sulfur in various fuels used in combustion engines. To improve manufacturing or optimise blending processes, it is desirable to monitor the sulfur content on-line with automatic analysis. Owing to the high number of hydrocarbons in the matrix and the possible wide variety of sulfur constituents, an individual determination of the sulfur constituents on-line is neither practical nor necessary. However, process analytical techniques performing total sulfur analysis must still meet several requirements including high sensitivity to sulfur, background matrix and sulfur species independence as well as long-term stability.

The described analytical system is utilising a new and unique system to vaporise small amounts of sample continuously. The completely vaporised sample is continuously burned in a flame to sulfur dioxide, water and carbon dioxide, where $SO_2$ represents the entire sulfur contents in the sample. After interference free separation of $SO_2$ using conventional gas chromatography, detection is achieved utilising a flame photometer detector.

This presentation will outline the analytical configuration, suitable applications, hardware and analytical challenges, results concerning sensitivity, linearity, long-term stability as well as analytical limitations determined.

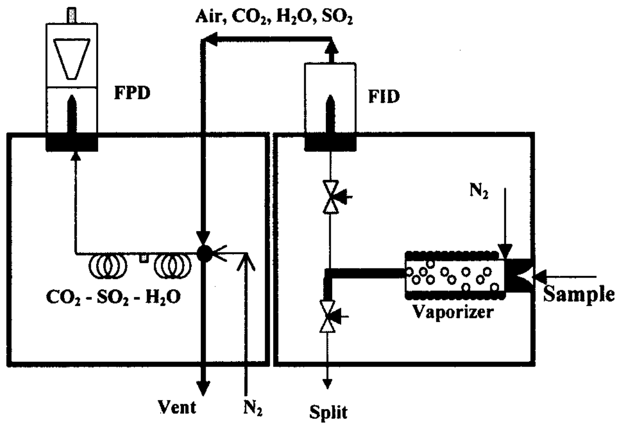

*System configuration with continuous vaporisation, conversion as well as separation and detection.*

### Speciation of mercury in organic matrices by gas chromatography coupled with a microwave-induced plasma atomic emission detector (GC-MIP-AED) and with an inductively coupled plasma mass spectrometry (GC-ICP-MS)

*Brice Bouyssiere[1], Franck Baco[2], Laurent Savary[3] and Ryszard Lobinski[1], [1]Laboratoire Chimie Analytique Bio-Inorganique et Environnement, UMR 5034, CNRS, Hélioparc Pau-Pyrénées, 2 av. Pierre Angot, F-64000 Pau, [2]Institut Français de Pétrole, CEDI 'René Navarre', BP 3, F-69390 Vernaison, [3]Institut Français de Pétrole, 164, av. Bois-Préau, F-92852 Rueil-Malmaison, France*

Speciation of mercury in organic matrices has attracted recently much interest especially due to corrosion and environmental risks in the petroleum industry. Challenges in developing reliable analytical protocols result from the presence of the hydrocarbon matrix which gives rise to background interferences and instability of the detector.

The paper discusses a number of approaches for speciation of polar organomercury compounds: MeHg, PrHg, $Hg_2$, EtHg and non-polar species: $Me_2Hg$, $Pr_2Hg$, $Bu_2Hg$, MePrHg and MeBuHg. The ability of the GC-ICP-MS to detect specifically the mercury vapour $Hg^{\circ}$ is demonstrated. The approaches are based on:

- direct analysis of a gas condensate by GC–ICP MS ($Hg^{\circ}$ and non-polar species);
- derivatization of the condensate by Grignard reaction followed by GC-MIP AES and GC-ICP MS (polar species); and
- extraction of mercury species by a cysteine solution followed by their re-extraction, derivatization and GC–MIP–AES (polar species, improved detection limits).

The figures of merit, advantages and shortcomings of the above approaches are critically compared.

The paper also discusses the rapid determination of the total mercury in gas condensates by ICP MS using cooled spray chamber, reduced diameter injector and the use of oxygen-rich plasma.

### Development of new instrumentation based on atomic fluorescence spectrometry for the speciation of mercury in natural gas

*A. Pisseloup[1], W. T. Corns[2], P. B. Stockwell[2], O. F. X. Donard[3] and O. Pecheyian[3], [1]Institut Français du Petrole, 1 et 4 Avenue de Bois-Priau, F-92852 Rueil Malmaison, Cedex, France, [2]P S Analytical Ltd, Arthur House, Crayfields Industrial Estate, Main Road, Orpington BR5 3HP, UK, [3]Laboratoire de Chimie Analytique Bio-Inorganique et Environnement, Helioparc, 2 Avenue du President Angot, F-64000 Pau, France*

The determination of mercury in natural gas is of major importance to the petrochemical industry. Mercury occurs natural in natural gas at concentrations between 0 and $200\,g\,m^{-3}$. The presence of mercury can have catastrophic effects on gas plants, even at low concentrations. These problems include mercury-induced corrosion on cryogenic aluminium heat exchangers, poisoning of hydrogenation catalysts and environmental pollution. The majority of measurements to date have focussed on the determination of total mercury, although more recently information on the speciation of mercury has been required to gain a better understanding of the mercury removal processes.

The development of novel sampling interfaces and instrumentation for the speciation of mercury in natural gas will be presented. Sampling techniques based on selective solid-phase adsorption of mercury species have been developed. Chromatographic materials such as Tenax were found to trap organomercury species quantatively, whereas elemental mercury was not retained. Gold impregnated onto silica, however, was found to collect all forms of mercury.

Placing the tubes in parallel or series therefore enabled speciation by difference. A thermal desorption system that can be coupled directly to atomic fluorescence spectrometry (AFS) to measure total mercury or organic mercury will be described.

Cryogenic focussing coupled to AFS has also been explored to identify the individual organomercury species trapped by Tenax. Data will be presented from both bench-scale and field applications.

### Environmental monitoring with glass powder

*Anja Krickser[1], Joachim Kuhn[1], Jochen Fricke[1] and Hannelore Roemich[2], [1]Bavarian Center for Applied Energy Research (ZAE Bayern), Am Hubland, 97074 Wuerzburg, [2]Fraunhofer-Institute for Silicate Research (ISC), Bronnbach Branch, Bronnbach 28, 97877 Wertheim/Bronnbach, Germany*

Potash lime silicate (PLS) glasses react with humidity and pollutants like sulphur dioxide, nitric oxides and organic acids. Synergetic effects are also observed. Sensors employing slices of such glasses are used to monitor the environmental impact on materials, e.g. on cultural heritage. This is realized by analysing the absorption band ~3 μm in the IR-transmission spectra before and after an exposure time of several months. The aim of this work is to increase the sensitivity of glass sensors. This is achieved by using a thin layer of PLS-glass powder on an IR-transparent organic substrate.

To correlate the reaction of the powder glass sensor with a defined climate, IR transmission measurements are performed before and after its exposure to the climate of interest. To monitor the transmission a FTIR spectrometer with a 1.8–18 μm spectral range in combination with an integrating sphere is employed.

Owing to the small optical thickness of the powder layer, the progress of corrosion can be detected not just at 3 μm (as is the case with glass slices) but at several additional bands. Thus, considerably more information can be obtained.

The progress of corrosion of the glass powder can be detected by observing the bands of silanol groups and molecular water, because the amount of these groups is increased by corrosion. There are two absorption bands of interest: the OH-stretching vibration at ~3 μm and the bending vibration of molecular water at ~6 μm.

As the intensity and the shape of these absorption peaks as well as the ratio of the intensity of the two peaks depend on the parameters responsible for the environmental impact on the sensor, detailed information can be extracted by observing the corrosion-induced changes of the 3 and 6 μm peaks. In addition, absorption peaks between 10 and 18 μm are investigated. These peaks are enhanced by the corrosion of the glass powder and are due to the structural changes in the surface layer of the glass (gel layer) and the corrosion crust consisting e.g. of sulphates and carbonates.

Our experiments show that different types of indoor and outdoor environments can be distinguished by their respective corrosivity levels already after just a few days of exposure. In our presentation, we report on the changes in directional-hemispherical transmission of PLS glass powders exposed to artificial and natural environments.

### Streamlining the dissolution test: automated HPLC techniques

*Kelly A. Johnson, Michael E. Swartz, Patricia A. Fowler and Charles H. Frasier, Waters Corp., Milford, MA, USA*

The fast pace of the modern pharmaceutical industry requires laboratories to reduce the analytical burden of their test procedures and increase productivity while still satisfying regulatory compliance. There are now several ways to meet these challenges in the dissolution laboratory.

Automated dissolution systems eliminate the slight variations that may occur in manual methods, thereby insuring reproducible data, higher throughput, and cost reduction. Validated single source software control of the entire automated system, as well as dissolution data acquisition and calculations, can further streamline the work flow while maintaining FDA compliance, as with 21 CFR Part 11. As a significant time and resource saver, the USP now approves several methods that involve the analysis of pooled dissolution samples in which individual dissolution aliquots are pooled and assayed as a single sample. Modern on-line HPLC dissolution systems can pool the individual samples and perform these types of analyses automatically, unattended. Similarly, the ability to automate sampling at shorter intervals and analyse a large number of samples via an on-line HPLC system may provide a more complete solution for the decision-making process in the early stages of drug development. Also, these systems must also be capable of handling increasingly complex formulations, such as multiple actives, widely varying dosages, as well as media types being used, such as buffers, organic solvents and surfactants.

We will present data generated by a single hardware and software source for a number of the above pharmaceutical dissolution applications, summarising a complete approach to reducing the analytical burden and increasing sample throughput in the dissolution laboratory.

### Advanced automation, system integrity, module and system validation, and maintenance in a new integrated high-performance liquid chromatograph

*Sebastian Farrell, Susan Steinike, Curtis Campbell, Masayuki Nishimura and John Monti, Shimadzu Scientific Instruments, Inc., 7102 Riverwood Drive, Columbia, MD 21046, USA*

Compliance with regulatory agencies as well as the need to maintain good laboratory and manufacturing standards is placing ever-increasing demands upon the modern analytical laboratory. This communication describes advanced features for automation, validation and maintenance in two newly designed, completely integrated high-performance liquid chromatographs. The Shimadzu LC-2010A and LC-2010C have the capability of automatic system preparation, system integrity monitoring, automatic start-up and shut-down, system and module validation, column monitoring and status reporting, and are also equipped with automated maintenance and problem notification features. The applicability and utility of these features in HPLC analyses will be discussed.

### LIMS in the age of e-commerce

*Christine Paszko and Tom Miller, Accelerated Technology Laboratories, Inc., 496 Holly Grove School Road, West End, NC 27376, USA*

E-commerce is increasingly gaining prominence and many companies have moved toward understanding how e-business can help them solve some of their business challenges.

Laboratories have come under increasing competitive pressures to reduce costs, increase profitability and increase their market share. There exists a large opportunity to sell complex products and services to a broader market over the Internet. The global nature of the Internet is spawning new business models for laboratories, changing business-to-business (B2B) relationships and increasing competitiveness. To date, the majority of the e-commerce activity has focused on the business to consumer market. However, today the fastest growing segment is believed to be the B2B sector. In April 1999, Forrester Research reported that B2B e-commerce sales reached $43.1 billion; their forecasts predict that by 2003, worldwide Internet sales will reach $3.2 trillion (with an annual growth rate of 99%). The focus of this presentation will be to understand the nature of the new models and their capabilities and how LIMS can be integrated into these new business models.

Analytical laboratories are responding to these changes to allow the sale of goods and services over the Internet. Laboratories are experimenting with new business models, selling goods and services over the Internet. The initial phase of navigating the Internet includes obtaining a Web presence, and setting up a site where clients can be educated on the goods and services that the laboratories offer, perhaps browse a catalog and select a product or service. Unlike simple products such as books, laboratory services are often more involved. There are two different types of complexity that arise; product or service complexity and needs complexity. Both will be addressed in this presentation.

### LIMS implementation: getting the best from your project team

*Nicholas J. Arnold, ThermoLabSystems, 1 St George's Court, Altrincham, Manchester WA14 5TP, UK*

As with many computer and other systems projects, the implementation of an LIMS can be a major undertaking for an organization. A successful implementation will depend on a number of factors with respect to both technology and people. A key factor will be the composition, formation and actions of a project team. This paper will address some of the issues involved in putting together the right project team; the development of the team; and the pressures, both internal and external, that the team might face during the project lifetime. The roles of individuals within the team will be examined from both the perspectives of technical skills needed, and of interpersonal behaviours required. The positions of team leader and project manager will also be discussed. Overall, the paper will attempt to identify and to categorize the project team issues that will have major bearing on a successful LIMS implementation.

### Web-based, workflow-oriented LIMS: a new dimension to your business

*Ulrika Soderlund and Johan Nor, N. Wilnor AB, Landerigrnden 4, SE-223 55 Lund, Sweden*

What is a workflow-oriented LIMS? Testing is an integrated part of a step-to-step production process. Each step of a test is controllable when it comes to, for example, order of processing, time and permission. In a workflow-oriented LIMS, each step of a SOP can be mirrored. All types of actions can be included, such as measurements or a verification that something has been performed, e.g. dissolution (see the figure).

How will it help me? A workflow-oriented LIMS makes it easy for a user to find information about what work to do and in which order to do it by visualizing the workflow. It can also be used when automation is required, since all steps, manual and automatic, can be documented. Managing the test procedure in a process-oriented way will bring higher efficiency and throughput together with improved traceability and quality assurance.

Ease of use is key. The use of well-known SOPs will bring evolution, not revolution, to your laboratory. Making use of a browser as interface, together with a highly configurable system, will cut the educational corners. The user interface is easy to navigate and will help users understand what and where to perform their tasks.

A workflow-oriented LIMS means increased efficiency with maintained quality. Why Web-based? Increased availability and true scalability is the answer. By employing Internet/Intranet solutions, an international corporation and even its customers and suppliers will be as readily available as your own computer. Modern Web-technology brings true scalability, the system can grow together with you while maintaining high performance.

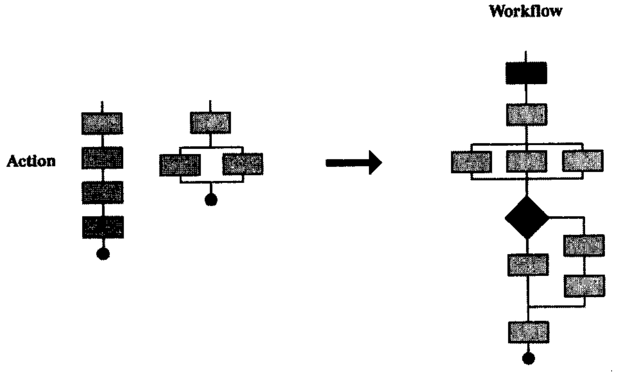

*Serial or parallel actions forms workflows to mirror your SOPs.*

**Water quality monitoring using a portable fiber-optic biosensor: RAPTOR**

*George P. Anderson, Naval Research Laboratory, Center for Bio/Molecular Science and Engineering, Code 6910, Washington, DC 20375-5348, USA*

The RAPTOR is a man-portable, automated biosensor capable of performing rapid, 10-min, assays on a sample for four target analytes simultaneously. It performs a fluorescent sandwich immunoassay on the surface of short polystyrene optical probes. The capture antibody is adsorbed to the probe surface, while fluorescently labeled antibodies are held in a separate reservoir.

Since target recognition is a two-step process, selectivity is enhanced, and the optical probes can be reused up to 40 times, or until a positive result is obtained. This greatly reduces the logistical burden for field operations. Numerous assays for toxins, such as SEB and ricin, and bacteria, such as *B. anthracis* and *F. tularensis*, have been developed for the RAPTOR. An assay of particular interest for water quality monitoring and the screening of fruits and vegetables is detecting the presence of *Giardia* cysts. *G. lambilia* is a parasitic protozoan that can cause a severe intestinal infection common in the developing world. Thus, a simple assay that could be used to screen water supplies while in the field would be highly useful. Such an assay has been developed using the RAPTOR. The detection limit for Giardia cysts was $5 \times 10^{-4}\,ml^{-1}$ for a 10-min assay.

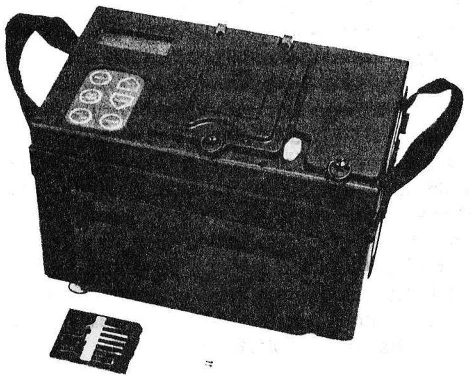

*RAPTOR fiberoptic biosensor.*

**Automated structure elucidation using a combination of NMR, MS and IR spectroscopy data**

*Antony J. Williams[1], Kirill Blinov[2], Eduard Martiroslan[2] and Mikhail Elyashberg[3], [1]Advanced Chemistry Development, 90 Adelaide Street West, Suite 702, Toronto, Ontario, M5H 3V9, Canada, [2]Advanced Chemistry Development, Russian Office, 6 Bakuleva Street, Moscow 117513, Russia, [3]All-Russian Research Institute of Organic Synthesis, 12 Radio Street, 107005, Moscow, Russia*

Automated structure elucidation commonly uses NMR spectroscopy as the foundation spectroscopic technique. We report here two approaches to the automated elucidation of structures from spectroscopic data. The first approach uses a one-dimensional C13 NMR spectrum in conjunction with H1 NMR, MS and IR data when available. Suggested structures are generated from fragment overlap using a fragment-based rules system commonly used in NMR prediction packages which have been successfully applied to the predictions of H1, C13, F19 and P31 spectra. Since any particular fragment has associated with it a set of C13 chemical shifts, and any spectrum is simply a superposition of spectral subsets with associated fragments, theoretically it is possible to extract suggested fragments with associated chemical shifts and build the full spectrum from appropriate overlap of these fragments. Using additional data including multiplicity data, quantitative intensities, H1 NMR spectral data, infrared spectral data, mass spectroscopic data and the molecular formula the probability of extracting appropriate fragments and generating the final structure is greatly increased.

Owing to the complexity of many chemical structures analysed by spectroscopy, it is uncommon to elucidate automatically the structure from standard one-dimensional spectra. Additional information from multidimensional NMR techniques is generally employed. The second automated elucidation approach, therefore, uses cross-peak responses from multidimensional NMR experiments. We will report here on our recent developments and work-in-progress to utilise two-dimensional spectroscopic data as part of the automated structure elucidation process.

**Air monitoring for phosphine using a pulsed flame-photometric detector**

*Sandra T. Macon, Donald P. Segers and Craig B. Lagrone, O. I. Analytical, CMS Field Products, 2148 Pelham, Parkway Building 400, Pelham, AL 35124, USA*

The Field MINICAMS®, manufactured by CMS Field Products, O.1. Analytical, is an automated air-monitoring system capable of detecting a wide range of volatile organic compounds. Phosphine, a widely used commercial fumigant, can be detected using the Field MINICAMS equipped with a pulsed flame-photometric detector (PFPD) and a sampling inlet configured with a gas-sampling loop.

Data will be presented demonstrating low, ppb detection of phosphine in air using short cycle times to provide continuous near-real-time MINICAMS monitoring for phosphine applications. The advantages of the PPFD for phosphine monitoring include high sensitivity, high selectivity to limit interferences and low operating gas consumption.

**Continuous sampling for low-level air-monitoring applications**

*Melody L. Thompson, Craig B. Lagrone, Kenneth A. Kuhn, Thomas J. McGuire and Donald P. SEGERS, O. 1. Analytical, CMS Field Products, 2148 Pelham, Parkway Building 400, Pelham, AL 35124, USA*

The Field MINICAMS®, manufactured by CMS Field Products, O. I. Analytical, is a field-portable ambient air-monitoring system designed for routine monitoring of low levels of volatile and semivolatile compounds, including chemical warfare (CW) agents and several toxic industrial compounds. When configured with optional hardware for continuous sample collection that overlaps with the gas-chromatographic analysis (instead of repetitive but non-continuous sampling-and-analysis cycles), report intervals for the compounds of interest can be decreased and gaps in sample collection are eliminated.

A dual-element, multiple-agent (VX, sarin and mustard) monitoring method incorporating continuous sampling has been developed for the pulsed flame-photometric detector (PFPD). The high volume continuous sampler (HVCS) provides for uninterrupted sampling of a specific environment, while allowing larger volumes of air to be collected for analysis. This is accomplished by the use of internal, redundant sample pathways and two preconcentrator tubes. The HVCS preconcentrator tubes are a greater diameter than those typically used with MINICAMS, consequently allowing larger volumes of air to be collected. While sample collection is taking place on one tube, the contents of the other tube are desorbed and refocused at the MINICAMS collection tube for subsequent chromatographic analysis. The desorbed tube is then purged and cooled so that it is ready for the next sampling cycle.

For the application presented, the HVCS provides several benefits. The overlapping sample-and-analysis time reduces the total cycle time/reporting interval and also eliminates any 'blind spots' in the monitoring. Larger sample volumes correlate to a higher measured response and, therefore, better precision and accuracy data.

**New approach for the verification of calibration for on-line TOC monitors**

*Ven A. Raman and Wisam Yacteen, Millipore Corp., Lab Water Division, 80 Ashby Road, Bedford, MA 01730, USA*

The detection and quantitation of organic contaminants in ultrapure water (UPW) has become increasingly important in several applications as well as in laboratories across multiple industries.

Most noteworthy today is the need for organic free water for several analytical measurements used in the environmental, pharmaceuticals, clinical and biological assays. The quality of organic free water is often designated by TOC (total organic/oxidisable carbon). In all the applications, the ultrapure water is used as solvent as well as blank for ultratrace analysis of low-level organic contaminants.

The most accepted method of detecting and measuring low-level organic contaminants in UPW is based on conductometric detection. Organic contaminants are oxidised (to $CO_2$) mediated by UV radiation and sometimes assisted by catalyst or oxidant.

These methods allow the measurement of all oxidisable organic substances and are designated by TOC. No other simple, reliable and economical method exists today for rapid on-line determination of TOC in ultrapure water. These methods rely on the change in conductivity produced by $CO_2$ before and after oxidation and, therefore, are calibrated using conductivity references rather than TOC reference. In other words, the TOC level is inferred from indirect measurements and verification is often done with instrument that suffers the same limitations as the instrument under calibration.

This paper presents an alternate approach that is simple and reliable that depends on independent measurement and does not rely on circular logic, and therefore, addresses one of the most serious concerns about the use of conductometric TOC measurements in critical applications.

Two direct independent methods were developed using simple commonly practiced UV and HPLC instruments. These approaches incorporate two separate calibration ranges capable of measuring low levels (2–50 ppb TOC) as well as high levels (50–1000 ppb) of TOC. The methods also used the USP 24(643) recommended marker compound, which is classified as theoretically difficult to oxidise by UV. Correlation between the TOC measured by indirect conductivity measurement and the UV and HPLC methods will be discussed.

**How reliable are continuous gas analysers?**

*Joachim A. Paulus and Carolyn Snyder, Siemens Applied Automation, 7101 Hollister Road, Houston, TX 77040, USA*

Infrared absorbing components measured by non-dispersive infrared (NDIR) radiation and oxygen determination based on paramagnetic properties are the most widespread types of continuous gas analysers. For several decades, their importance for both process and emission measurement has been unrivaled.

Reliability is surely not a scientific term and it is obvious that several different parameters contribute to the reliability of an analyser. A comparison of the readings with reference methods, the cross-sensitivity against other components or the availability over a given period along with other measurements can be seen as characteristic for a certain model of analyser.

The paper presents such data for the newest generation of analysers with lower measuring ranges and improved features. Data were gathered by a manufacturer-

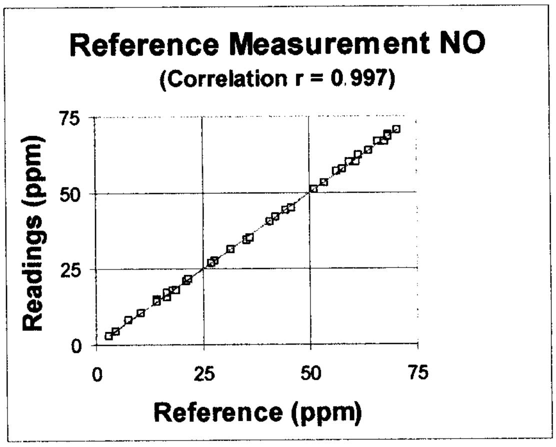

*Correlation of readings (analyser 4) with reference methods (CLD I wet chemistry).*

independent institution in both field and in-house tests utilising standardised methods.

Six gas analysers were operated for some weeks in a laboratory and for ~4 months in a waste incineration. The evaluation shows excellent overall compliance with regulations given by authorities and also with the expectations of customers.

### Trace quantitative analysis in a process FTICR

*Steve C. Beu, Jyh-Shing Chen, Dean V. Davis, Jim Klahn, Alex Mintskovsky and Richard Thompson, Applied Automation, Inc., 500 West Highway 60, Bartlesville, OK 74003, USA*

The ideal trace analyser should be simple, sensitive, universal, fast, specific, durable, low-maintenance, quantitative and capable of identifying unknowns. Mass spectrometers come closest to satisfying all of these criteria. Their main weaknesses in the past have been their durability, simplicity and maintenance requirements. They also occasionally suffer from lack of specificity and must be coupled with some form of separation, which greatly effects their response time. A new process mass spectrometer, the Quantra, has been developed that addresses some of these weaknesses using an ion cyclotron resonance platform for mass analysis rather than a quadrupole or magnetic sector.

The ICR is more versatile as its mass range is three to four times greater than current process MS alternatives. It is more specific due to its well-known mass measurement accuracy coupled with its higher resolution. An example of which is included in the figure. It is not as sensitive as it does not incorporate a multiplier for detection. It also is not operated in a single-ion monitoring mode. This can either be an advantage or disadvantage depending on whether specificity or sensitivity is more important to a given problem. In addition, the Quantra is an extremely versatile instrument with CI, negative ion, and spectral manipulation capability built in. This adds a variety of target analytes beyond traditional process MS capabilities.

Actual examples from real-world problem solving will used to clarify and illustrate these capabilities.

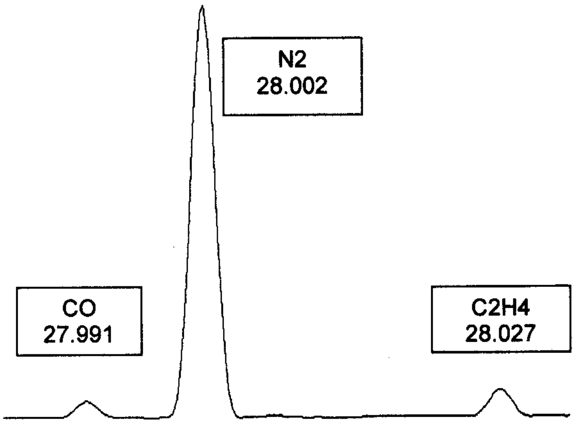

*Three peaks at 28, resolution 12 800.*

### Moving double-focusing mass spectrometry into the industrial process monitoring environment

*Mark E. Koslin, Larry McDermott, John Irlam, Bruce McIntosh and Marty Ranc, Orbital Sciences Corp., 2771 N. Garey Avenue, Pomona, CA 91767, USA*

Mass spectrometry has been used for industrial process monitoring for over 25 years and double-focusing mass spectrometers have been used in the laboratory environment for many decades. The introduction of a double-focusing, magnetically scanned mass spectrometer designed specifically for industrial process monitoring was made in 2000. The MGA iSCAN design marries the ruggedness and reliability of military and space instrumentation with the 'workhorse' performance of the MGA 1200EC.

The following iSCAN features provide the production manager with a powerful tool to optimise his process.

- Continuous detection range from 10 ppb to 100% with a SIN ratio >3:1.
- Analyse for reactive gases.
- Unity resolution across the mass range 2–200 amu.
- Ability to analyse for up to 40 compounds.
- Typical analysis time <1 min.
- Analytical drift of <0.05% of reading per day.
- Direct software control of multipoint sampling system for up to 50 different sample locations.
- Software control of sample site sequencing.
- Ability to detect unknown process constituents.

Detailed mass spectra are available for off-line analysis. Over the years, magnetic sector-based mass spectrometers have demonstrated significant stability advantages over other types of mass spectrometers like quadrupole-based system. But single focusing, magnetic sector mass spectrometers have also been physically larger than quadrupole-based systems to achieve the same resolution. A double-focusing (energy and momentum) design eliminates this disadvantage. The resulting compact design offers a package that easily fits into industrial environment. In fact, the MGA iSCAN has the same footprint of predecessors resulting in an easy upgrade path for existing customers.

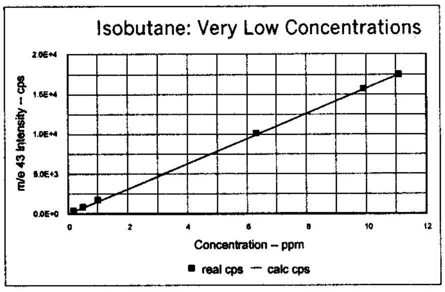

*MGA iSCAN's linearity over the range 170–11 ppm for isobutane.*

